# A modelling framework to characterize the impact of antibiotics on the gut microbiota diversity

**DOI:** 10.1080/19490976.2024.2442523

**Published:** 2024-12-22

**Authors:** Carlos Olivares, Etienne Ruppé, Stéphanie Ferreira, Tanguy Corbel, Antoine Andremont, Jean de Gunzburg, Jeremie Guedj, Charles Burdet

**Affiliations:** a Université Paris Cité, IAME, INSERM, Paris, France; b APHP, Laboratoire de Bactériologie, Hôpital Bichat, Paris, France; c Da Volterra, Paris, France

**Keywords:** Human gut microbiota, antibiotics, metagenomics, pharmacokinetics, pharmacodynamics, nonlinear mixed effect modeling

## Abstract

Metagenomic sequencing deepened our knowledge about the role of the intestinal microbiota in human health, and several studies with various methodologies explored its dynamics during antibiotic treatments. We compared the impact of four widely used antibiotics on the gut bacterial diversity. We used plasma and fecal samples collected during and after treatment from healthy volunteers assigned to a 5-day treatment either by ceftriaxone (1 g every 24 h through IV route), ceftazidime/avibactam (2 g/500 mg every 8 h through IV route), piperacillin/tazobactam (1 g/500 mg every 8 h through IV route) or moxifloxacin (400 mg every 24 h through oral route). Antibiotic concentrations were measured in plasma and feces, and bacterial diversity was assessed by the Shannon index from 16S rRNA gene profiling. The relationship between the evolutions of antibiotic fecal exposure and bacterial diversity was modeled using non-linear mixed effects models. We compared the impact of antibiotics on gut microbiota diversity by simulation, using various reconstructed pharmacodynamic indices. Piperacillin/tazobactam was characterized by the highest impact in terms of intensity of perturbation (maximal [IQR] loss of diversity of 27.3% [1.9; 40.0]), while moxifloxacin had the longest duration of perturbation, with a time to return to 95% of baseline value after the last administration of 13.2 d [8.3; 19.1]. Overall, moxifloxacin exhibited the highest global impact, followed by piperacillin/tazobactam, ceftazidime/avibactam and ceftriaxone. Their AUC between day 0 and day 42 of the change of diversity indices from day 0 were, respectively, −13.2 Shannon unit.day [−20.4; −7.9], −10.9 Shannon unit.day [−20.4; −0.6] and −10.1 Shannon unit.day [−18.3; −4.6]. We conclude that antibiotics alter the intestinal diversity to varying degrees, both within and between antibiotics families. Such studies are needed to help antibiotic stewardship in using the antibiotics with the lowest impact on the intestinal microbiota.

## Background

The human intestinal microbiota constitutes a complex ecological system made of an estimated 10^13^ of microorganisms, including bacteria, viruses, fungi, and archaea.^1^ These diverse organisms interact with each other and with the host to form a delicate balance influencing the overall well-being of the host and contributing to essential metabolic, immunological, and neurological functions.^2^

Antibiotic treatments are among the most significant disruptors of the microbiota due to their intrinsic antibacterial mechanism. Whether administered orally or parenterally, antibiotic residues are eliminated via the digestive tract, disrupting the balance of intestinal microbiota. Short-term consequences of this perturbation may include antibiotic-associated diarrhea, *Clostridioides difficile* enteritis and selection of resistant bacteria. Antibiotic use has also been associated with poor outcomes in patients with allogeneic hematopoietic-cell transplantation.^3^ Long-term consequences may also occur, including perturbation of the metabolic and immune functions. The administration of antibiotics is shortly followed by the reduction of the bacterial diversity within the intestinal ecosystem,^4,5^ which is believed to have significant implications for human health. Indeed, several studies suggested a link between intestinal bacterial diversity and the risk of developing certain diseases. For instance, low levels of bacterial diversity have been observed in patients with inflammatory bowel diseases and type 2 diabetes, indicating that the loss of bacterial diversity may be associated with an increased susceptibility to metabolic and inflammatory diseases.^6,7^ In a recent paper, β-lactam antibiotics were reported to decrease the efficacy of immune checkpoint inhibitors in a preclinical cancer model.^8^ The reduction of fecal concentrations of β-lactam antibiotics by an active charcoal-based adsorbent was shown to restore their efficacy.

A comprehensive and quantitative understanding of the relationship between antibiotic administration and the disruption of the intestinal microbiota is thus crucial for guiding the use of antibiotics and minimizing their adverse effects.^9^ While the clinical spectrum refers to the minimum inhibitory concentration for a given pathogenic bacterium and the antibiotic concentration obtained at a given infected site, the impact on the gut microbiota depends on the concentrations achieved in the gut content, and on the susceptibility of the components of the bacterial consortia forming the gut microbiota and its diversity. This latter is still not well characterized, as many bacteria from the intestinal microbiota cannot be characterized *in vitro* and were identified using high throughput sequencing methods. It is thus plausible that antibiotic impact on the gut microbiota is not fully superposable to their clinical spectrum.^10^ Pharmacokinetic and pharmacodynamic modeling provides a valuable framework for quantifying the relationship between antibiotic concentrations and bacterial diversity, considering inter-individual variabilities and temporal dynamics of the perturbation. Mathematical modeling can indeed be used to simulate various antibiotic treatment scenarios, such as different dosages, or durations to predict their specific impacts on the microbiota, forecasting long-term impacts based on short-term data. It thus allows to compare the effect of antibiotics on microbial diversity and helps identify antibiotics that cause less disruption. Such analytical frameworks are currently lacking.

Here, we used the data from the DAV132-CL-1006 clinical trial^8^ and modeled the relationships between the evolution of plasma and fecal concentration of three antibiotics from the β-lactam family (ceftriaxone, piperacillin/tazobactam, and ceftazidime/avibactam) and that of bacterial diversity in the intestinal microbiota, applying a previously developed methodological framework.^11,12^ Subsequently, we compared, through simulations, the effects of these three antibiotics and moxifloxacin on intestinal microbiota diversity.

## Material and methods

### Healthy volunteers and sample collection

We used the data from DAV-132-CL1006, a single center, prospective, randomized, controlled, parallel groups, repeated doses, open-label phase 1 clinical trial sponsored by DaVolterra (Paris, France). The trial aimed at assessing the effect of DAV132, a charcoal-based adsorbent designed to adsorb antibiotic residues in the late ileum and gut,^13^ on plasma concentrations of β-lactams. It was conducted at Biotrial, Rennes, France, between May 2019 and December 2019. The trial obtained approval from the Independent Ethics Committee Sud-Ouest et Outre-Mer 1 on 20 May 2019 and from the National Agency for Security of Medicinal Products on 07 May 2019 and was conducted with respect to good clinical practice and the Declaration of Helsinki as last amended. Full results of the trial have been reported elsewhere.^8^

Eligible subjects were healthy adults, aged 18–60 years old, with a body mass index (BMI) between 18.5 and 30 kg/m^2^ and a normal digestive transit. Subjects were excluded if they had any history of gastrointestinal disorder, hospitalization, or antibiotic administration within 3 months prior to inclusion. None of them presented *Clostridioides difficile* carriage at inclusion. All subjects received oral and written information and provided signed consent before inclusion. The full list of inclusion and exclusion criteria is presented in Supplementary Text S1.

**Figure 1.**
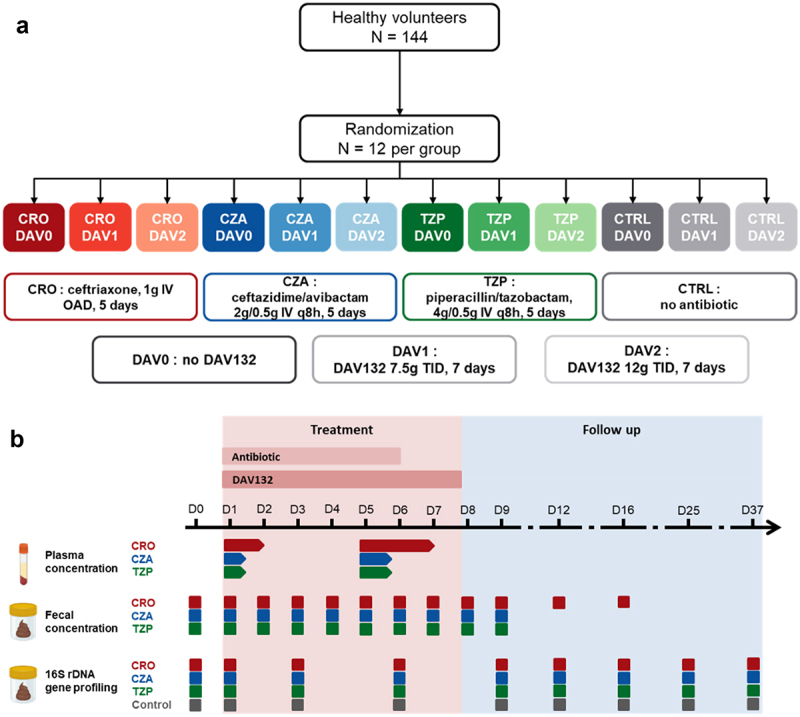
Design of the DAV132-CL-1006 clinical trial.Prospective randomized clinical trial including healthy volunteers into one of 12 groups of treatment (panel A): no ATB control (CTRL) without and with DAV132, CRO, CZA or PTZ alone, CRO, CZA or PTZ in combination with DAV132. Antibiotics were administered by the intravenous route for 5 d, and DAV132 was administered orally for 7 d. Several plasma and fecal samples were collected for determination of fecal concentrations of antibiotics and fecal microbiota analysis (panel B). CRO, Ceftriaxone; CZA, Ceftazidime/avibactam; TZP, Piperacillin/tazobactam; MXF, Moxifloxacin; q8h, every 8 hours; OAD, every 24 hours; TID, three times a day.

A total of 144 subjects were included and randomized in a 1:1 ratio to one of twelve arms: DAV132 7.5 g tid, or DAV132 12 g tid, or No DAV132, combined with a defined β-lactam group (ceftriaxone, CRO, 1 g every 24 h, or piperacillin/tazobactam, TZP, 4 g/0.5 g every 8 h, or ceftazidime/avibactam, CZA, 2 g/0.5 g every 8 h) (12 subjects/group) or no beta-lactam. DAV132 was administered orally for 7 d, and antibiotics were administered intravenously for 5 d. Day 1 was defined as the first day of antibiotic treatment. The design of the trial is summarized in the Figure 1.

For each subject, several blood samples were collected at day 1 and day 5 for determination of the total concentration of antibiotics in plasma. Blood samples were collected in the CRO group on day 1 (just before, 0.25, 0.5, 1, 2, 4, 6, 8, 12, 18, 24 h after the start of infusion on day 1 and) and day 5 (just before, 0.25, 0.5, 1, 2, 3, 4, 6, 8, 24, 36, 48 h after the start of infusion) of treatment, for TZP on day 1 (just before, 0.25, 0.5, 0.75, 1, 1.5, 2, 3, 4, 6, 8 h after the start of infusion) and day 5 (just before, 0.25, 0.5, 0.75, 1, 1.5, 2, 3, 4, 6, 8, 12 and 18 h after the start of infusion). For CZA on day 1 (just before, 1, 1.5, 2, 2.5, 3, 4, 6, 8 after the start of infusion) and day 5 (just before, 1, 1.5, 2, 2.5, 3, 4, 6, 8, 12, 18 after the start of infusion) of treatment. The exact times of the beginning and end of infusion, as well as the exact sampling times, were recorded. Blood samples were taken from the arm opposite that used for antibiotic administration.

Stool samples were collected before the beginning of treatment, daily between day 1 and day 9, and at days 12, 16, 25 and 37. Antibiotic concentration in feces were measured in samples collected up to day 9, as well as at days 12 and 16 for the CRO group.

Ceftriaxone plasma concentrations were measured using a LC-MS/MS method after sample preparation by protein precipitation. The lower limit of quantification (LLOQ) in plasma was 0.05 µg/mL. For feces, the assay comprised sample preparation by liquid-liquid extraction and subsequent analysis of the extract by LC-MS/MS. The LLOQ in feces was 0.04 µg/g.

Piperacillin and tazobactam plasma concentrations were measured using a LC-MS/MS method after sample preparation by protein precipitation. The LLOQ in plasma were 0.05 µg/mL for both piperacillin and tazobactam. For feces, the assay comprised sample preparation by free fraction collection followed by liquid–liquid extraction and subsequent analysis of the extract by LC-MS/MS. The LLOQs were 0.04 µg/g for both piperacillin and tazobactam.

Ceftazidime and avibactam plasma concentrations were measured using a LC-MS/MS method after sample preparation by protein precipitation. The LLOQ in plasma were 0.04 and 0.0098 µg/mL for ceftazidime and avibactam, respectively. For feces, the assay comprised sample preparation by dilution of acidified feces filtrate samples for ceftazidime and by heating and dilution of feces filtrate samples for avibactam, followed by subsequent analysis of the extract by LC-MS-MS. The LLOQs in feces were 0.04 µg/g for both ceftazidime and avibactam.

### Targeted metagenomic analysis of the intestinal microbiota

Samples obtained just before the beginning of treatment and at days 3, 6, 9, 12, 16, 25 and 37 were analyzed by 16S rRNA gene profiling for the groups treated by beta-lactams and the No DAV132/no beta-lactam control group.

The extraction of the bacterial DNA was performed using an in-house protocol partially based on the QIAamp® DNA stool Kit, (Qiagen). It included several in-house lysis steps, which combined chemical and mechanical methods.

The V3 and V4 regions of the 16S rRNA gene were amplified using the Metabiote amplicon library preparation protocol, standardized and optimized by GenoScreen (Lille, France). This protocol includes both negative (sterile water) and positive (artificial community of 17 bacteria) controls.

PCRs were performed for 30 cycles using genomic DNA (5 ng), in-house fusion primers (0.2 M) and an annealing temperature of 50°C. Agencourt AMPure XP magnetic beads (Beckman Coulter, CA, USA) were used to purify PCR products, which were then quantified using GenoScreen’s internal protocol. Sequencing was performed using the Illumina MiSeq platform (Illumina, CA, USA) at GenoScreen (Lille, France).

Raw paired-end reads were then demultiplexed. Primers were then removed using CutAdapt^14^ without any mismatch. A Phred quality score of 30 was used for quality filtering performed with PRINSEQ-lite PERL.^15^ Paired-end reads were then assembled using FLASH^16^ and the following parameters: with a minimum overlap of ≥30 bases and 97% identity.

Estimation of bacterial diversity in each sample was performed using GenoScreen’s Metabiote Online v2.0 pipeline (Lille, France), based on QIIME v1.9.1.^17^ Chimera sequences were eliminated using an in-house method based on Usearch v6.1,^18^ and DNA sequences were clustered (97% identity threshold) with Uclust v1.2.22q^18^ through an open-reference OTU picking process and the complete-linkage method. Finally, OTU singletons were suppressed, and diversity was estimated by computing the Shannon diversity index for each fecal sample. The Shannon diversity index (SI) is a dimensionless index, computed as: $\mathrm{SI}=-\overset{n}{\underset{i=1}{\sum }}p_{i}\times \mathrm{lo}g_{2}(p_{i})$, where $p_{i}$ is the relative abundance of the OTU i and n is the number of OTUs detected in each fecal sample.

### Modelling strategy

We first developed models for the pharmacokinetics of antibiotic. Then, we accounted for DAV132 administration and effect on the fecal concentrations of antibiotics. Finally, we modeled the effect of fecal antibiotic concentration on the bacterial diversity. This approach was based on.^11,12,19^

#### Pharmacokinetic model for the antibiotics

We first developed compartmental models of plasma antibiotic concentrations, using total drug concentrations from all antibiotic-treated groups. Each antibiotic was modeled separately, and one- and two-compartment models with first-order elimination were tested.

Then, each antibiotic-specific model was extended to fecal pharmacokinetics. The fecal weight was fixed to 200 g/day for every subject, and we restricted this model-development step to groups receiving antibiotics only. Elimination of the antibiotic from the central compartment was split into first-order rates intestinal and extra-intestinal elimination. We tested several models with various numbers of transit compartments between the central and fecal compartments.

#### Model for the kinetic of DAV132 and antibiotic adsorption on the active charcoal

We then used a model previously developed by our team to model the disposition of DAV132 following administration and the release of the active charcoal in the lower gastro-intestinal tract over time.^12^ All antibiotics-treated groups were analyzed simultaneously. For the adsorption of fecal antibiotic by the active charcoal, we used a linear model where the amount of bound fecal antibiotic is proportional to the amount of activated charcoal in the lower gastro-intestinal tract.^12^

The evolution of the amount of activated charcoal in the lower gastrointestinal tract was thus described using the following system of ordinary differential equations:

$$\frac{dA_{a1}^{DAV132}}{\mathrm{dt}}=\mathrm{Dos}e^{\mathrm{DAV}132}-k_{t}^{DAV132}\times A_{a1}^{DAV132}$$

$$\frac{dA_{a2}^{DAV132}}{\mathrm{dt}}=k_{t}^{DAV132}\times A_{a1}^{DAV132}-k_{t}^{DAV132}\times A_{a2}^{DAV132}$$

$$\frac{dA_{a3}^{DAV132}}{\mathrm{dt}}=k_{t}^{DAV132}\times A_{a2}^{DAV132}-k_{t}^{DAV132}\times A_{a3}^{DAV132}$$

$$\frac{dA_{f}^{C\mathrm{harc}}}{\mathrm{dt}}=k_{t}^{DAV132}\times A_{a3}^{DAV132}-k_{f}^{Ch\mathrm{arc}}\times A_{f}^{Ch\mathrm{arc}}$$

where $\mathrm{Dos}e^{\mathrm{DAV}132}$ is the unit dose of DAV132; $A_{a1}^{DAV132}$, $A_{a2}^{DAV132}$ and $A_{a3}^{DAV132}$ are the amounts of DAV132 in the first, second and third transit delivery compartments; $A_{f}^{Ch\mathrm{arc}}$ is the amount of activated charcoal in the lower gastro-intestinal tract; $k_{t}^{DAV132}$ is the transit delivery rate of DAV132 and $k_{f}^{Ch\mathrm{arc}}$ is the elimination rate constant of activated charcoal.

#### Pharmacodynamic model of the antibiotic effect on bacterial diversity

Finally, we used a model of indirect pharmacodynamic response to describe the impact of the antibiotic on the bacterial diversity.^20^ The evolution of the diversity index SI in the absence of drug was written as:

$$\frac{\mathrm{dSI}}{\mathrm{dt}}=R_{\mathrm{in}}-k_{\mathrm{out}}\times \mathrm{SI}$$

where $R_{\mathrm{in}}$ is a zero-order constant for the production of diversity, and $k_{\mathrm{out}}$ is a first-order rate constant for its elimination. At steady state, we assume that the Shannon diversity index is constant, hence $\frac{\mathrm{dSI}}{\mathrm{dt}}=0$, thus in the absence of treatment $SI_{0}=\frac{R_{\mathrm{in}}}{k_{\mathrm{out}}}$. We assumed that antibiotic treatment perturbs this equilibrium and increases $k_{\mathrm{out}}$ as:

$$k_{out}^{\prime }=\left(1+\frac{E_{\max}\times C_{f}}{EC_{50}+C_{f}}\right)\times k_{\mathrm{out}}$$

where $C_{f}$ is the free fecal antibiotic concentration, $E_{\max}$ is the maximal increase of $k_{\mathrm{out}}$ by the antibiotic, and $EC_{50}$ is the concentration leading to 50% of $E_{\max}$. β-lactamase inhibitor were assumed to be deprived of any effect, and data from the control group were added (with fecal concentrations assumed to be 0 in the control group).

### Statistical model

Nonlinear mixed effects models were used to analyze the pharmacokinetic and pharmacodynamic data. Let $y_{i}$ be the vector of observations for all responses for the individual i and $y_{\mathrm{ik}}$ the vector of observations for the $k^{\mathrm{th}}$ response. f denotes the global structural model characacterizing all responses, similar for all individuals. Then in individual i, $y_{\mathrm{ik}}=f_{k}\left(\theta_{i},\xi_{\mathrm{ik}}\right)+\varepsilon_{\mathrm{ik}}$, where $f_{k}$ is the component of fdescribing the $k^{\mathrm{th}}$ repsonse, $\theta_{i}$ is the vector of individual parameters, $\xi_{\mathrm{ik}}$ is the vector of $n_{\mathrm{ik}}$ sampling times and $\varepsilon_{\mathrm{ik}}$ is the vector of residual errors for the $k^{\mathrm{th}}$ response. Each individual parameter $\theta_{i}$ can be decomposed as a fixed effect μ, representing the mean parameter value in the population, and a random effect $b_{i}∼N\left(0,\Omega \right)$ (with Ω accounting for interindividual variability). Assuming an exponential random effect model, individual parameters are modeled as: $\theta_{i}=\mu \times e^{b_{i}}$.

We assumed that the residual errors are distributed as $\varepsilon_{\mathrm{ik}}∼N\left(0,\Sigma_{\mathrm{ik}}\right)$ where $\Sigma_{\mathrm{ik}}$ is a $n_{\mathrm{ik}}\times n_{\mathrm{ik}}$-diagonal matrix with $k^{\mathrm{th}}$ elements equal to ${\left(\sigma_{\mathrm{inter},k}+\sigma_{\mathrm{slope},k}\times f_{k}\left(\theta_{i},\xi_{\mathrm{ik}}\right)\right)}^{2}$. $\sigma_{\mathrm{inter},k}$ is the parameter for the additive part and $\sigma_{\mathrm{slope},k}$ is the parameter for the proportional part of the variance error model. Constant ($\sigma_{\mathrm{inter},k}$ ≠ 0, $\sigma_{\mathrm{slope},k}$ = 0), proportional ($\sigma_{\mathrm{inter},k}$ = 0, $\sigma_{\mathrm{slope},k}$ ≠ 0) or combined ($\sigma_{\mathrm{inter},k}$ ≠ 0, $\sigma_{\mathrm{slope},k}$ ≠ 0) variance error models were tested for each response k.

### Parameters estimation

Estimation of population parameters was performed using the stochastic approximation expectation maximization algorithm (SAEM),^21^ implemented in Monolix 2018R2 and 2021R2, a software devoted to parameters estimation in mixed effect nonlinear modeling (Lixoft, France, www.lixoft.eu). Data below the lower limit of quantification were treated as left-censored data. Their contribution to the likelihood was computed as the probability that these data are indeed below the lower limit of quantification.^22^

First, we modeled the evolution of antibiotics concentrations in plasma and feces as previously described. Then, individual pharmacokinetic profiles were predicted using empirical Bayes estimates. Individual predicted fecal concentrations were used in the analysis of the diversity indices, and the diversity was assumed to be stable in the absence of treatment.

For random effects, in case of low estimated standard deviation (<0.1) and high relative standard error, parameter variability was set to 0.

### Model selection and evaluation

At each step, the best model was chosen using the corrected Bayesian information criteria (BICc), derived for each model from the likelihood computed by importance sampling with 2.10^5^ iterations. Model evaluation was conducted by investigating several goodness-of-fit plots: individual fits, plots of predictions versus observations, distribution of the individual weighted residuals (IWRES) and normalized prediction distribution errors (NPDE) versus time and versus model predictions, as well as the visual predictive check (VPC). NPDE and VPC were generated using 500 Monte Carlo simulations.

### Measures of antibiotic impact on the fecal content

To get a better sense of the evolution of fecal antibiotic concentrations over time and to quantify the effect of antibiotics on the gut microbiota diversity, we simulated 1000 pharmacokinetic and pharmacodynamic profiles at steady state for each antibiotic in the asymptotic distribution of the parameters, using the simulX 2021R2 package in R (R Foundation for Statistical Computing, Vienna, Austria). In order to enrich the analysis and allow comparisons across antibiotic families, we also simulated profiles for moxifloxacin, a fluoroquinolone antibiotic which was previously studied in healthy volunteers using the same methodology than the one used in the present analysis (see reference^12^). Antibiotic daily doses were 1 g OAD for ceftriaxone, 4 g/0.5 g q8h for piperacillin/tazobactam, 2 g/0.5 g q8h for ceftazidime/avibactam, and 400 mg q24h for moxifloxacin. The following treatment durations were used: 3, 5, 7, 10 and 14 d. For each simulated subject and each antibiotic, we computed the maximal fecal concentration achieved and the time needed after the last dose to decrease below the limit of quantification. The following pharmacodynamic indices were computed: maximal loss of bacterial diversity in the intestinal microbiome after the beginning of treatment (nadir), time for which this maximal loss was achieved (time to nadir) and time at which each diversity index returned to 95% of its baseline value. The cumulated impact of each antibiotic on diversity was obtained by computing the area under the curve for each diversity index up to 42 d after the beginning of treatment.

## Results

Among the 121 participants included in the present analysis (12 from the control group, 36 from the CZA group, 36 from the CRO group and 37 from the TZP group), the median age of participants included in the modeling analysis was 36 y (min; max 18; 60) and 64 were males (52.9%). Median weight was 71.6 kg (49; 95).

### Pharmacokinetic and pharmacodynamic modeling

For the pharmacokinetic model, a total of 1029 to 1285 values of concentrations were available for each drug (see Supplementary Figure S1-S3). For all drugs, the plasma concentrations were best described by a two-compartment model with first-order elimination. Compartmental models including 3 to 4 transit compartments between plasma and feces best fitted the fecal data. Mathematical expressions of the models are provided in Supplementary Material (Supplementary Text S2).

Parameters estimate of the pharmacokinetic models could be estimated with a good precision, most of them having a residual standard error below 30%, with the exception of parameters related to piperacillin disposition in feces (Supplementary Tables S1-S3). The adsorption of the beta-lactam on the active charcoal varied according to the antibiotic. It was lowest for ceftriaxone (4.4.10^−5^/mg.h) and highest for piperacillin (0.32/mg.h). Avibactam was found to be poorly adsorbed by the charcoal.

The pharmacokinetic models of each antibiotic well characterized the evolution of the fecal concentrations over time, both for beta-lactam antibiotics and co-administered beta-lactamase inhibitors. Individual fits, along with other goodness-of-fit plots, are provided in the Supplementary Figures S4-S15.

For the pharmacodynamic modeling, a total of 925 samples were analyzed for bacterial diversity estimation (CRO, 278 samples; CZA, 279 samples; TZP, 274 samples; Ct, 94 samples). The administration of antibiotic lead to a rapid drop of the Shannon index (Supplementary Figure S16), which slowly returned to pre-treatment values after treatment cessation. The dynamics of the Shannon index following the beginning of the antibiotic treatment was well captured by our model, and goodness-of-fit plots were satisfactory (Supplementary Figures S17-S18). All parameters were well estimated (Supplementary Table S4), except for the piperacilin EC_50_. This might be related to the high estimated interindividual variability of this parameter. The bacterial diversity of the gut was differentially affected following antibiotic administration, in relation to different estimates of susceptibility parameters. The first-order elimination rate of the Shannon index was increased by 78% (r.s.e., 1.0%) with piperacillin, by 58.0% (22.4%) with ceftazidime and by 7.1% (39.1%) with ceftriaxone. The concentration leading to 50% of the maximal effect of antibiotics was 6.68 µg/g (122.8%) for piperacillin, 10.90 µg/g (23.7%) for ceftazidime and 0.15 µg/g (18.9%) for ceftriaxone.

### Simulations of fecal antibiotic concentrations and gut diversity

To get a better sense of the impact of antibiotic administration on gut bacterial diversity, and compare their respective effects, we evaluated by simulation the evolution of the Shannon diversity index with standard doses of either ceftriaxone, ceftazidime/avibactam, piperacillin/tazobactam, or moxifloxacin, for durations of 3–14 d, without DAV132 administration. We previously conducted a similar modeling analysis of moxifloxacin data,^12^ and included it in the simulations in order to enrich the comparison of antibiotics impact on the gut bacterial diversity with another antibiotic family.

**Figure 2.**
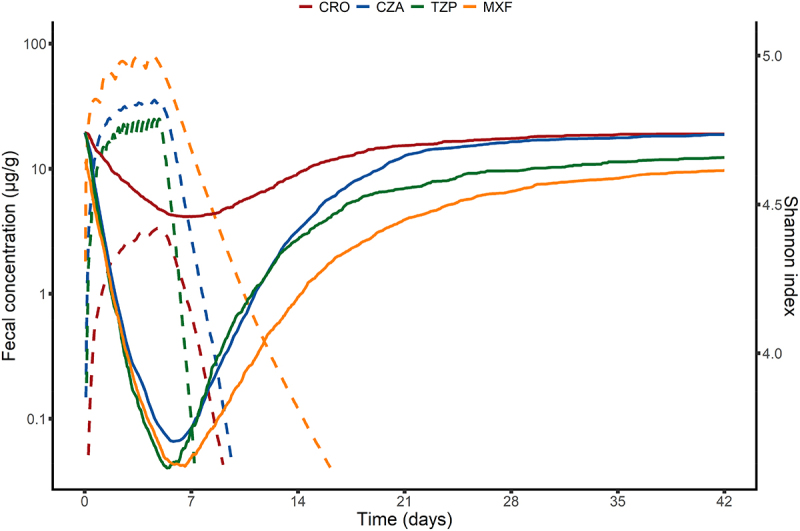
Median profiles of fecal antibiotic concentrations and of the Shannon diversity index following a 5-day treatment. This figure depicts the evolution over time of the fecal concentrations of ceftriaxone, ceftazidime/avibactam, piperacillin/tazobactam and moxifloxacin, and of the Shannon diversity index after administration of these antibiotics in human healthy volunteers. Results were obtained for each antibiotic from 1000 individuals simulated in the asymptotic distribution of the parameters estimated in the final model. Plain lines depict the evolution of the Shannon index, while dashed lines depict the evolution of fecal antibiotic concentrations. CRO, Ceftriaxone; CZA, Ceftazidime/avibactam; TZP, Piperacillin/tazobactam; MXF, moxifloxacin.

The evolution of the fecal concentration of antibiotics, along with the evolution of the Shannon index following a 5-day treatment is depicted in Figure 2.

In the pharmacokinetic simulations, moxifloxacin had the highest maximal concentration achieved in feces (80.7 µg/g [62; 109.2] following a 5-day treatment), and ceftriaxone had the lowest (3.5 µg/g [0.6; 23.4] following a 5-day treatment) (Figure 2 and Supplementary Table S5). The time to decrease below the limit of quantification was highest for moxifloxacin (12.2 d after the last administration following a 5-day treatment [10.8; 13.6]), being more than 2-times higher than that of ceftriaxone and ceftazidime (Supplementary Table S5). It was the lowest for piperacillin (2.5 d [1.4; 5]).

**Figure 3.**
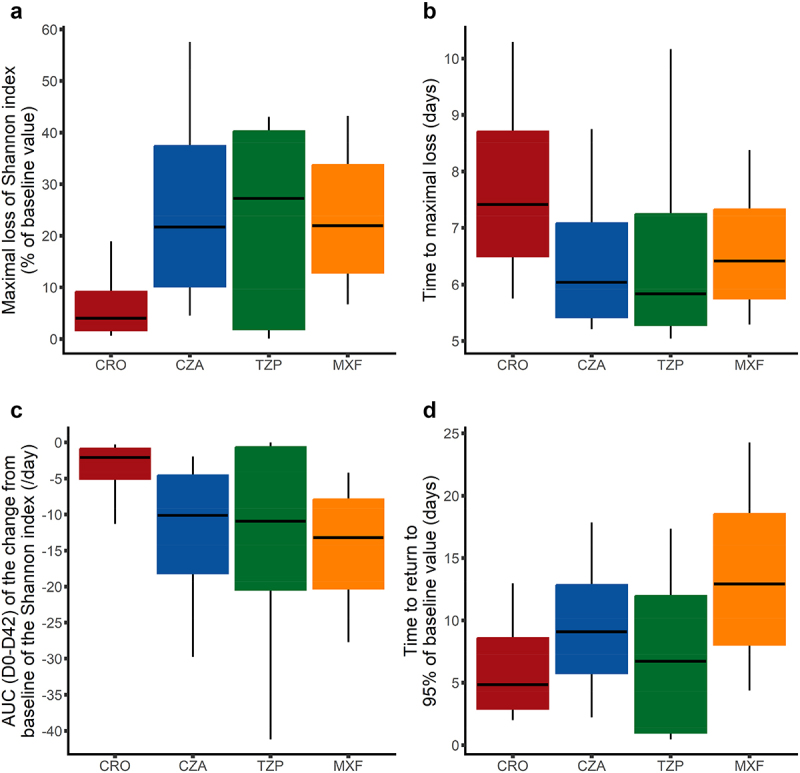
Derived indices of the impact of antibiotics on the bacterial gut microbiota following a 5-day treatment. Results were obtained for each antibiotic from 1000 individuals simulated in the asymptotic distribution of the parameters estimated in the final model. The boxes present the 25th and 75th percentiles and the horizontal black bar reports the median value, while whiskers report 10th and 90th percentiles. CRO, Ceftriaxone; CZA, Ceftazidime/avibactam; TZP, Piperacillin/tazobactam; MXF, moxifloxacin.

In the pharmacodynamic simulations, ceftriaxone exerted the lowest disturbance on the intestinal microbiota, both in terms of intensity (median maximal loss of 4% of baseline value [IQR, 1.7%; 9.1%] following a 5-day treatment) and time to return to 95% of the baseline value of 4.0 d [1.7; 9.1] after the last administration. As a result, the overall impact of ceftriaxone administration was the lowest of the four antibiotics analyzed (Figure 3 and Supplementary Table S5). Other antibiotics were differentiated by a variable ranking according to the pharmacodynamic index analyzed. Taking a 5-day treatment as an illustration, piperacillin/tazobactam had the highest impact in terms of intensity of perturbation, with a maximal loss of 27.3% [1.9%; 40%] of the Shannon index baseline value, while moxifloxacin had the longest duration of perturbation, with a time to return to 95% of baseline value of 17.8 [13.0; 24.0] d after the last administration. Overall, results of our simulations showed that moxifloxacin exhibited the highest global impact on the Shannon diversity index, as measured by the AUC between day 0 and day 42 of the change from baseline of the Shannon index, followed by piperacillin/tazobactam, ceftazidime/avibactam and ceftriaxone (Figure 3). Regarding the maximal loss of Shannon diversity index, piperacillin/tazobactam had the highest impact, followed by moxifloxacin, ceftazidime/avibactam and ceftriaxone.

**Figure 4.**
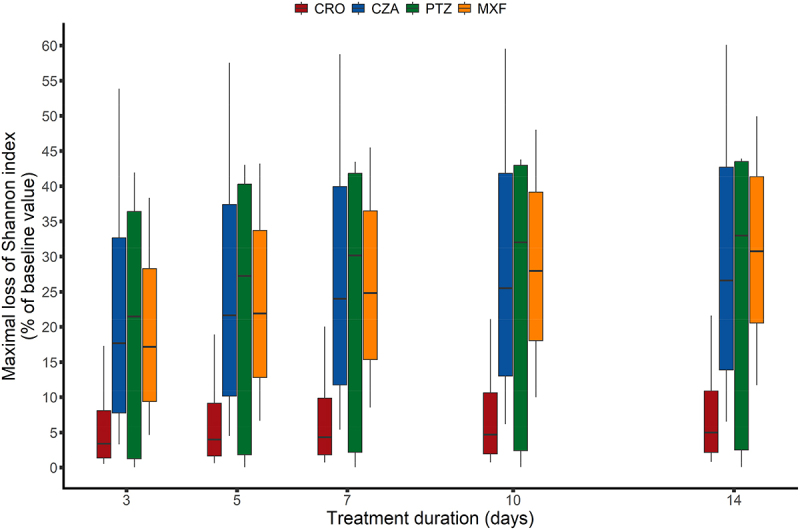
Maximal loss of Shannon index according to antibiotic treatment and to its duration. Results were obtained for each antibiotic from 1000 individuals simulated in the asymptotic distribution of the parameters estimated in the final model. The boxes present the 25th and 75th percentiles and the horizontal black bar reports the median value, while whiskers report 10th and 90th percentiles. CRO, Ceftriaxone; CZA, Ceftazidime/avibactam; TZP, Piperacillin/tazobactam; MXF, moxifloxacin.

As for the evolution of the impact according to antibiotic treatment duration, we observed that the impact increased with treatment duration to various extent (Figure 4 and Supplementary Table S5 and Supplementary Figures S19-S22). Both the maximal loss of the Shannon index relative to baseline and the AUC of the change from baseline of the Shannon index between days 0 and 42 increased in a non-linear fashion with treatment duration: doubling treatment duration from 5 d to 10 d (100% increase) did not result in doubling the impact on the microbiota but increased it to a lower extent (57%−76% depending on the antibiotic). In the same way, reducing the treatment duration from 5 to 3 d (40% relative reduction) would result in the reduction of approximately 30% of the global impact on gut microbiota diversity. The time to maximal loss was approximately 2 d higher than treatment duration, and the time for the Shannon index to return to 95% of its baseline value did not vary with treatment duration.

## Discussion

While recent advances in metagenomic sequencing revealed that the gut microbiota exert an unsuspected role in various host processes, antibiotic administration has been reported to have a disruptive impact on its equilibrium. Here, we adapted previously developed mathematical models^12^ to characterize the relationship between fecal levels of three β-lactam antibiotics and the temporal dynamics of gut bacterial diversity during and after treatment initiation.

This allowed to perform Monte Carlo simulations to compare the impact on the gut microbiota of these 3 antibiotics, to which we added the fluoroquinolone moxifloxacin, which was used in the previously developed model.^12^

We observed that moxifloxacin, piperacillin/tazobactam and ceftazidime/avibactam exerted a marked effect on the gut bacterial diversity, while the effect of ceftriaxone was more limited.

Pharmacokinetic analysis showed that piperacillin exhibited the quickest elimination from the fecal compartment after the last administration: its fecal concentration decreased below the fecal limit of quantification approximately 2.5 d after the beginning of the last administration. It is interesting to notice that this time is still much longer than the time required for the plasma concentration to decrease below the limit of quantification (about 1 h^23^). Despite this rapid elimination, piperacillin/tazobactam exhibited the most profound impact on gut bacterial diversity among studied β-lactams, with a reduction by 27% of the baseline value. Its maximal effect on gut bacterial diversity (78% increase of the elimination of bacterial diversity) was the highest among the studied β-lactams, and a little below that of moxifloxacin (83% increase of the elimination of bacterial diversity^12^). This profound impact of piperacillin/tazobactam association is probably related to its broad-spectrum activity, against both aerobic and anaerobic bacteria.

In line with its highest $E_{\max}$ value, moxifloxacin exhibited the highest global impact on gut bacterial diversity, as measured by the area under curve of the change from baseline of the Shannon index between days 0 and 42. This can be explained as the high maximal effect of moxifloxacin on the gut bacterial diversity (−21.9% of the baseline value for a 5-day treatment) was associated with a slow return of gut bacterial diversity to baseline value after cessation of antibiotic administration (approximately 18 d after a 5-day treatment). This slow return might be related to the slow decrease of the moxifloxacin fecal concentration and to the enteric recirculation of the antibiotic.^24^

On the other side, ceftriaxone exhibited a low impact on gut bacterial diversity, with a 5 to 6 times lower global impact than for the other studied antibiotics. The elimination of ceftriaxone from the fecal content required a median time of 5.1 d, ranging between piperacillin and moxifloxacin, and this lower impact was concomitant with a relatively small impact on the maximal loss of diversity following treatment initiation (−4% of the baseline value).

In the field of antibacterial resistance, international and national organisms defined classifications of antibiotics to preserve their effectiveness and limit the risk of toxicity.^25,26^ Our study demonstrates how modeling could complement specific research on bacterial resistance to classify antibiotics and provide synthetic information for optimizing their use when multiple alternatives are available. In the future such approaches could be used to individualize antimicrobial therapy. Interestingly, we observed that after a single course of antibiotics, the Shannon diversity index went back to baseline values within a few weeks. This is different than what was shown by precursor studies in healthy subjects repeatedly treated by ciprofloxacin, an fluoroquinolone antibiotic.^4,27^ This might be related to a shift in the gut microbiota composition, whose return to baseline values of bacterial diversity might mask underlying, pervasive effects with a switch in bacterial composition that persist when antibiotic treatments are repeated. This underlines the need for further studies that includes thorough analysis of the perturbation, including a functional analysis.

In the absence of existing specific studies, it is difficult to determine whether the results obtained using metagenomic sequencing methods would be comparable to those obtained through traditional bacterial culture methods. It is, however, widely acknowledged that these four antibiotics have an important impact on the gut microbiota, reducing the counts of Enterobacterales and Bifidobacteriales and, for the 3 β-lactam antibiotics, of lactobacilli.^28^ Our approach, using the Shannon index – a synthetic index of bacterial diversity associated with pathologic states – allows to get a more global evaluation of the drug effect, taking into account the whole composition of the bacterial community that constitutes the microbiota.

This modeling approach is also useful to perform simulations and predict the expected effect of various treatment regimens. Here, we could predict that doubling the treatment duration from 5 d to 10 d would not result in doubling the impact on the microbiota but in increasing the impact to a lower extent: the global impact would increase by 1.6 and the maximal loss of diversity by 1.2. This suggests that the deleterious effect in reducing the gut bacterial diversity occurs quickly after treatment initiation and that efforts should be made to develop diagnostic tools for quickly identify which patients require antibiotic initiation for a bacterial infection. This nonlinear relationship between the impact of antibiotics and treatment duration might be explained by the initial elimination of sensitive bacteria, causing a sharp drop in microbial diversity. This initial reduction disrupt ecological balances and could trigger cascading effects where other bacteria are affected as well. Over time, the remaining community would thus become increasingly dominated by resistant bacteria, making the microbiota less susceptible to further antibiotic effects. This front-loaded impact results in a dramatic early response that tapers off as treatment continues.

Protecting the gut from the deleterious effects of antibiotics should thus include the reevaluation of recommended antibiotic treatment durations, when possible. This question regained interest in recent years, and several recent clinical trials investigated the efficacy of shortened treatment durations, for instance for the treatment of community-acquired pneumonia in clinically stable patients admitted to the hospital (with results suggesting that a 3-day treatment is non-inferior to 7-day treatment in patients with community-acquired pneumonia who met clinical stability criteria)^29^ or in men with a febrile urinary tract infection (with results suggesting that 7-day treatment duration is inferior to 14-day treatment).^30^

Our work has some limitations. First, this study was performed using data obtained from healthy volunteers, while the studied antibiotics are mainly administered for serious infections in hospitalized patients. Antibiotic pharmacokinetics is known to be modified in infected patients, with modifications of the drug’s volume of distribution or clearance, which might modify elimination parameters and the achieved concentration dynamics in the gut.^31^ In addition, the composition of the gut microbiota might be different in such patients, who often present comorbid conditions. The modifications brought by sepsis on the fecal elimination of antibiotics, and on possible changes on the gut microbiota, are still not well characterized, and should be further studied. Meanwhile, we believe that such studies aiming at comparing the impact of antibiotics on the gut bacterial microbiota should primarily focus on healthy volunteers, as they allow avoiding these confounding factors that might bias the evaluation, and should further be confirmed in patients. This, however, makes harder the study the effect of antibiotics on the emergence of resistant bacterial strains, as included subjects must not have been treated by antibiotics in the months preceding enrollment in the trial. Second, some parameters could not be obtained with a good precision. To circumvent this limitation, we performed a high number of Monte Carlo simulations in order to be able to cover the entire distribution of population parameters, and for each drug we applied the same picked pharmacokinetic and pharmacodynamic parameters to alternate treatment durations. Second, we could not evaluate the effect of the DAV132 charcoal on bacterial diversity, as data were not available for modeling. It was, however, previously shown that DAV132 had no effect on the gene bacterial richness of the fecal content, making plausible the assumption of the absence of DAV132 effect on the bacterial diversity.^32^ Third, the follow-up duration was somehow limited to 37 d. Although this does probably not impact our analysis of the Shannon diversity index as the time to return to 95% of the baseline value was around 13 d, this might prevent to have complete perspective on the gut microbiota changes during and after treatment for other microbiota endpoint. Further studies should take into account this aspect and include longer follow-up. Last, we restrained our analysis on the Shannon index, and did not model the effect on the individual taxa obtained through metagenomic sequencing. Several hundreds of individual taxa were indeed identified, restraining the possibility of a deep analysis of the impact of the antibiotics on each specific taxa.

In summary, we applied here a modeling framework to measure the impact of various antibiotics on the intestinal microbiota and extended it to several β-lactam antibiotics. We were able to estimate several pharmacodynamic parameters to assess the impact of these antibiotics on the gut bacterial diversity, and to compare these impacts. We found that the fluoroquinolone moxifloxacin had an important effect in reducing gut bacterial diversity, while that of ceftriaxone appeared quite limited. This encourages to extend this approach to other antibiotics and gut microbiota markers, in order to inform clinicians when they can choose among several agents for treating infected patients. Future studies should investigate whether these observations are confirmed in patients, whose previous exposition to antibiotics or health conditions might influence the response of the microbiota to an antibiotic perturbation. They should also include a deeper analysis of the gut microbiota, including functional aspects, to better characterize the effect of antibiotics.

## Supplementary Material

Supplementary_Appendix_20241108_clean.docx

## Data Availability

The 16S rRNA gene profiling data are available from the European Nucleotide Archive under accession number PRJNA922086 (www.ebi.ac.uk/ena/browser/view/PRJNA922086). The clinical data will be made available upon request to the corresponding author.

## References

[1] Lozupone CA, Stombaugh JI, Gordon JI, Jansson JK, Knight R. Diversity, stability and resilience of the human gut microbiota. Nature. 2012;489(7415):220–14. doi:10.1038/nature11550. 22972295 PMC3577372

[2] Hou K, Wu Z-X, Chen X-Y, Wang J-Q, Zhang D, Xiao C, Zhu D, Koya JB, Wei L, Li J, et al. Microbiota in health and diseases. Signal Transduct Target Ther. 2022;7(1):135. doi:10.1038/s41392-022-00974-4. 35461318 PMC9034083

[3] Peled JU, Gomes ALC, Devlin SM, Littmann ER, Taur Y, Sung AD, Weber D, Hashimoto D, Slingerland AE, Slingerland JB, et al. Microbiota as predictor of mortality in allogeneic hematopoietic-cell transplantation. N Engl J Med. 2020;382(9):822–834. doi:10.1056/NEJMoa1900623. 32101664 PMC7534690

[4] Dethlefsen L, Relman DA. Incomplete recovery and individualized responses of the human distal gut microbiota to repeated antibiotic perturbation. Proc Natl Acad Sci USA. 2011;108(Supplement_1):4554–4561. doi:10.1073/pnas.1000087107. 20847294 PMC3063582

[5] Burdet C, Grall N, Linard M, Bridier-Nahmias A, Benhayoun M, Bourabha K, Magnan M, Clermont O, d’Humières C, Tenaillon O, et al. Ceftriaxone and cefotaxime have similar effects on the intestinal microbiota in human volunteers treated by standard-dose regimens. Antimicrob Agents Chemother U States. 2019;63(6). doi:10.1128/AAC.02244-18. PMC6535507 30936104

[6] Le Chatelier E, Nielsen T, Qin J, Prifti E, Hildebrand F, Falony G, Almeida M, Arumugam M, Batto J-M, Kennedy S, et al. Richness of human gut microbiome correlates with metabolic markers. Nature. 2013;500(7464):541–546. doi:10.1038/nature12506. 23985870

[7] Frank DN, St AA, Feldman RA, Boedeker EC, Harpaz N, Pace NR. Molecular-phylogenetic characterization of microbial community imbalances in human inflammatory bowel diseases. Proc Natl Acad Sci USA. 2007;104(34):13780–13785. doi:10.1073/pnas.0706625104. 17699621 PMC1959459

[8] Messaoudene M, Ferreira S, Saint-Lu N, Ponce M, Truntzer C, Boidot R, Le Bescop C, Loppinet T, Corbel T, Féger C, et al. The DAV132 colon-targeted adsorbent does not interfere with plasma concentrations of antibiotics but prevents antibiotic-related dysbiosis: a randomized phase I trial in healthy volunteers. Nat Commun. 2024;15(1):8083. doi:10.1038/s41467-024-52373-8. 39278946 PMC11402973

[9] Ruppé E, Burdet C, Grall N, de Lastours V, Lescure F-X, Andremont A, Armand-Lefèvre L. Impact of antibiotics on the intestinal microbiota needs to be re-defined to optimize antibiotic usage. Clin Microbiol Infect Off Publ Eur Soc Clin Microbiol Infect Dis. England; 2018;24(1):3–5. doi:10.1016/j.cmi.2017.09.017. 28970162

[10] Woerther P-L, d’ HC, Lescure X, Dubreuil L, Rodriguez C, Barbier F, Fihman V, Ruppé E. Is the term “anti-anaerobic” still relevant? Int J Infect Dis. 2021;102:178–180. doi:10.1016/j.ijid.2020.10.052. 33127500

[11] Burdet C, Nguyen TT, Duval X, Ferreira S, Andremont A, Guedj J, Mentré F, Ait-Ilalne B, Alavoine L, Duval X, et al. Impact of antibiotic gut exposure on the temporal changes in microbiome diversity. Antimicrob Agents Chemother U States. 2019;63(10). doi:10.1128/AAC.00820-19. PMC6761552 31307985

[12] Guk J, Guedj J, Burdet C, Andremont A, de Gunzburg J, Ducher A, Mentré F. Modeling the effect of DAV132, a novel Colon-targeted adsorbent, on fecal concentrations of moxifloxacin and gut microbiota diversity in healthy volunteers. Clin Pharmacol Ther U States. 2021;109(4):1045–1054. doi:10.1002/cpt.1977. 32617960

[13] de Gunzburg J, Ducher A, Modess C, Wegner D, Oswald S, Dressman J, Augustin V, Feger C, Andremont A, Weitschies W, et al. Targeted adsorption of molecules in the colon with the novel adsorbent-based medicinal product, DAV132: a proof of concept study in healthy subjects. J Clin Pharmacol. 2015;55(1):10–16. doi:10.1002/jcph.359. 25042595

[14] Martin M. Cutadapt removes adapter sequences from high-throughput sequencing reads. EMBnet J. 2011;17(1):10. doi:10.14806/ej.17.1.200.

[15] Schmieder R, Edwards R. Quality control and preprocessing of metagenomic datasets. Bioinformatics. 2011;27(6):863–864. doi:10.1093/bioinformatics/btr026. 21278185 PMC3051327

[16] Magoč T, Salzberg SL. FLASH: fast length adjustment of short reads to improve genome assemblies. Bioinforma Oxf Engl. 2011;27(21):2957–2963. doi:10.1093/bioinformatics/btr507. PMC3198573 21903629

[17] Caporaso JG, Kuczynski J, Stombaugh J, Bittinger K, Bushman FD, Costello EK, Fierer N, Peña AG, Goodrich JK, Gordon JI, et al. QIIME allows analysis of high-throughput community sequencing data. Nat Methods. 2010;7(5):335–336. doi:10.1038/nmeth.f.303. 20383131 PMC3156573

[18] Edgar RC. Search and clustering orders of magnitude faster than BLAST. Bioinformatics. 2010;26(19):2460–2461. doi:10.1093/bioinformatics/btq461. 20709691

[19] Burdet C. Impact d’une antibiothérapie sur le microbiote intestinal [Internet]. Paris: Université Sorbonne Paris Cité; 2018. https://theses.hal.science/tel-02167851/.

[20] Dayneka NL, Garg V, Jusko WJ. Comparison of four basic models of indirect pharmacodynamic responses. J Pharmacokinet Biopharm. 1993;21(4):457–478. doi:10.1007/BF01061691. 8133465 PMC4207304

[21] Kuhn E, Lavielle M. Maximum likelihood estimation in nonlinear mixed effects models. Comput Stat Data Anal. 2005;49(4):1020–1038. doi:10.1016/j.csda.2004.07.002.

[22] Samson A, Lavielle M, Mentré F. Extension of the SAEM algorithm to left-censored data in nonlinear mixed-effects model: application to HIV dynamics model. Comput Stat Data Anal. 2006;51(3):1562–1574. doi:10.1016/j.csda.2006.05.007.

[23] Sörgel F, Kinzig M. Pharmacokinetic characteristics of piperacillin/tazobactam. Intensive Care Med. 1994;20(3):S14–20. doi:10.1007/BF01745246. 7962984

[24] Stass H, Kubitza D, Möller J-G, Delesen H. Influence of activated charcoal on the pharmacokinetics of moxifloxacin following intravenous and oral administration of a 400 mg single dose to healthy males. Br J Clin Pharmacol. 2005;59(5):536–541. doi:10.1111/j.1365-2125.2005.02357.x. 15842551 PMC1884843

[25] Sharland M, Pulcini C, Harbarth S, Zeng M, Gandra S, Mathur S, Magrini N. Classifying antibiotics in the WHO essential medicines list for optimal use—be AWaRe. Lancet Infect Dis. 2018;18(1):18–20. doi:10.1016/S1473-3099(17)30724-7. 29303731

[26] Budd E, Cramp E, Sharland M, Hand K, Howard P, Wilson P, Wilcox M, Muller-Pebody B, Hopkins S. Adaptation of the WHO essential medicines list for national antibiotic stewardship policy in England: being AWaRe. J Antimicrob Chemother. 2019;74(11):3384–3389. doi:10.1093/jac/dkz321. 31361000

[27] Dethlefsen L, Huse S, Sogin ML, Relman DA, Ja E, Eisen JA, editor. The pervasive effects of an antibiotic on the human gut microbiota, as revealed by deep 16S rRNA sequencing. PLoS Biol. 2008;6(11):e280. doi:10.1371/journal.pbio.0060280. 19018661 PMC2586385

[28] Bhalodi AA, van ET, Virk HS, Wiersinga WJ. Impact of antimicrobial therapy on the gut microbiome. J Antimicrob Chemother. 2019;74(Suppl 1):i6–i15. doi:10.1093/jac/dky530. 30690540 PMC6382031

[29] Dinh A, Ropers J, Duran C, Davido B, Deconinck L, Matt M, Senard O, Lagrange A, Makhloufi S, Mellon G, et al. Discontinuing β-lactam treatment after 3 days for patients with community-acquired pneumonia in non-critical care wards (PTC): a double-blind, randomised, placebo-controlled, non-inferiority trial. Lancet Lond Engl Engl. 2021;397(10280):1195–1203. doi:10.1016/S0140-6736(21)00313-5. 33773631

[30] Lafaurie M, Chevret S, Fontaine J-P, Mongiat-Artus P, de Lastours V, Escaut L, Jaureguiberry S, Bernard L, Bruyere F, Gatey C, et al. Antimicrobial for 7 or 14 days for febrile urinary tract infection in men: a multicenter noninferiority double-blind, placebo-controlled, randomized clinical trial. Clin Infect Dis Off Publ Infect Dis Soc Am. 2023;76(12):2154–2162. doi:10.1093/cid/ciad070. 36785526

[31] Pea F, Viale P, Furlanut M. Antimicrobial therapy in critically ill patients: a review of pathophysiological conditions responsible for altered disposition and pharmacokinetic variability. Clin Pharmacokinet. 2005;44(10):1009–1034. doi:10.2165/00003088-200544100-00002. 16176116

[32] de Gunzburg J, Ghozlane A, Ducher A, Le Chatelier E, Duval X, Ruppé E, Armand-Lefevre L, Sablier-Gallis F, Burdet C, Alavoine L, et al. Protection of the human gut microbiome from antibiotics. J Infect Dis U States. 2018;217(4):628–636. doi:10.1093/infdis/jix604. PMC5853327 29186529

