# Supplementary material for: A modelling framework to characterize the impact of antibiotics on the gut microbiota diversity
Source: Gut Microbes. 2024 Dec 22;17(1):2442523. doi: 10.1080/19490976.2024.2442523 (PMC12931723; doi:10.1080/19490976.2024.2442523)
Supplement: Supplementary_Appendix_20241108_clean.docx [file KGMI_A_2442523_SM1297.docx]

Supplementary Appendix

# Supplementary Text S1 – Inclusion and exclusion criteria Inclusion criteria

Subjects will be required to satisfy all the following criteria to be included in the study:

1. Healthy adults (males and females), able to read and write, aged from 18 to 60 years old, inclusive.
2. Body mass index (BMI) between 18.5 and 30 kg/m², inclusive.
3. Considered as healthy individuals according to a comprehensive clinical assessment (detailed medical history and complete physical examination).
4. Vital signs evaluated after 10 minutes in normal supine position: systolic blood pressure between 95 and 140 mmHg, diastolic blood pressure between 45 and 90 mmHg, heart rate between 40 and 100 bpm (all results should be normal or, if out of range, non-clinically significant as determined by the investigator).
5. Normal digestive transit, with usually at least one daily stool.
6. Females participating in the study:
   - Either must be of non-child bearing potential (surgically sterilized at least 3 months prior to inclusion, or postmenopausal). Menopause is defined as being over 60 years of age, or between 45 and 60 years of age and having amenorrhea for at least 2 years with known plasma FSH level >30 IU/L;
   - or, if of child bearing potential, must have a negative urine pregnancy test within 7 days prior to receiving the first dose of the study treatment and should not be breastfeeding at screening, and must use abstinence or a double contraception method during the treatment period and for an additional period of 2 weeks after the end of investigational treatment. The accepted double contraception methods include the use of a highly effective method of birth control (intrauterine device or hormonal contraception) in addition to one of the following contraceptive options: (1) condom; (2) diaphragm or cervical/vault cap; or (3) spermicide.
7. Having given and signed the written study informed consent prior to undertaking any study-related procedure.

# Exclusion criteria

Subjects presenting any of the following criteria will not be included in the study: Exclusion criteria related to the health status

1. Any history or presence of clinically relevant cardiovascular, pulmonary, gastrointestinal, hepatic, renal, metabolic, hematological, neurological, bone and joint, muscular, psychiatric, systemic, ocular, gynecologic (if female), or infectious disease, or signs of acute illness.
2. Any history of relevant gastro-intestinal acute transient disorders within three months prior to the inclusion (e.g. colitis or gastritis, infective diarrhea, chronic diarrhea of unknown etiology, chronic constipation, celiac disease).
3. History of cholecystectomy.
4. Frequent headaches and/or migraine, recurrent nausea and/or vomiting (for each event, more than twice a month). Subject suffering from migraine at D1 will be excluded.

Exclusion criteria specific to the study

1. Presence or history of beta-lactams (penicillin or cephalosporin) hypersensitivity, or allergic disease diagnosed and treated by a physician; history of recurrent rashes, or any contra-indication to betalactams.
2. Contra-indications to charcoal, or risk factors for AEs associated to charcoal other than those already included into the inclusion/exclusion criteria: known hypersensitivity to charcoal, risk of GI obstruction, digestive perforation or digestive hemorrhage, decreased bowel sounds, abdominal distension, ileus.
3. Recent history of hospitalization (within the last 3 months).
4. Any antibiotic administration within the last 3 months.
5. Any vaccination within the last 28 days
6. Fecal colonization by C. difficile: PCR assay positive for C. difficile at the screening visit.
7. Subjects using probiotics indicated for the gut flora protection and refusing to stop them at inclusion and during the study.
8. Subjects currently taking activated charcoal.

Exclusion criteria associated to the pharmacokinetic evaluations

1. Blood donation, regardless of the volume, within 2 months before inclusion and during the study.
2. Intake of medications or herbal products within 14 days before inclusion, with the exception of episodic intakes of paracetamol or antispasmodic or chronic intake of hormonal contraception or menopausal hormone replacement therapy

Exclusion criteria associated to addiction

1. History or presence of alcohol abuse (alcohol consumption more than 40 g per day), or positive alcohol test.
2. Smoking more than 5 cigarettes or equivalent per day (including nicotine -delivering devices such as patches, gums and electronic cigarettes), unable to stop smoking during the study.
3. History or presence of drug abuse, or positive result on urine drug screen (amphetamines/methamphetamines, barbiturates, benzodiazepines, cannabinoids, cocaine, opiates)

Exclusion criteria related to administrative issue

1. Any subject who, in the judgment of the Investigator, is likely to be noncompliant during the study, or unable to cooperate.
2. Any subject in the exclusion period of a previous biomedical research according to national law, and participation to any other clinical trial simultaneously.
3. Any subject who cannot be contacted in case of emergency.
4. Any subject who belongs to the Investigating Center staff.
5. Subject of legal age unable of giving consent.
6. Subject deprived of liberty by judicial or administrative decision.
7. Subject of legal age under legal protection.
8. Subjects not covered by the French health insurance system or by another health insurance system where applicable and in compliance with the recommendations of the French law in force relating to biomedical research.
9. Subjects under administrative or legal supervision.

# Supplementary Text S3 – Final pharmacokinetic models

Ceftriaxone pharmacokinetic model

The evolution of the amount of ceftriaxone in the various compartments was described using the following system of ordinary differential equations:

𝑑𝐴𝐶𝑅𝑂

𝑐 = 𝑘𝐶𝑅𝑂 × 𝐴𝐶𝑅𝑂 − (𝑘𝐶𝑅𝑂 + 𝑘𝐶𝑅𝑂 + 𝑘𝐶𝑅𝑂) × 𝐴𝐶𝑅𝑂

𝑑𝑡

21 𝑝

𝑑𝐴𝐶𝑅𝑂

𝑒 𝑐𝑡 12 𝑐

𝑝 = 𝑘𝐶𝑅𝑂 × 𝐴𝐶𝑅𝑂 − 𝑘𝐶𝑅𝑂 × 𝐴𝐶𝑅𝑂

𝑑𝑡 12 𝑐

𝑑𝐴𝐶𝑅𝑂

21 𝑝

𝑡1 = 𝑘𝐶𝑅𝑂 × 𝐴𝐶𝑅𝑂 − 𝑘𝐶𝑅𝑂 × 𝐴𝐶𝑅𝑂

𝑑𝑡

𝑑𝐴𝐶𝑅𝑂

𝑐𝑡 𝑐 𝑡

𝑡1

𝑡2 = 𝑘𝐶𝑅𝑂 × 𝐴𝐶𝑅𝑂 − 𝑘𝐶𝑅𝑂 × 𝐴𝐶𝑅𝑂

𝑑𝑡 ^𝑡^

𝑑𝐴𝑡𝑎𝑧

𝑡1 𝑡

𝑡2

𝑡3 = 𝑘𝐶𝑅𝑂 × 𝐴𝐶𝑅𝑂 − 𝑘𝐶𝑅𝑂 × 𝐴𝐶𝑅𝑂

𝑑𝐴𝐶𝑅𝑂

𝑑𝑡 ^𝑡^

𝑡2 𝑡

𝑡3

𝑓 = 𝑘𝐶𝑅𝑂 × 𝐴𝐶𝑅𝑂 − 𝑘𝐶𝑅𝑂 × 𝐴𝐶𝑅𝑂 − 𝑘𝐶𝑅𝑂 × 𝐴𝑐ℎ𝑎𝑟𝑐 × 𝐴𝐶𝑅𝑂

𝑑𝑡 ^𝑡^

𝑡3 𝑓

𝑓 𝑎𝑑 𝑓 𝑓

where 𝐴^𝐶𝑅𝑂^ is the amount of ceftriaxone in the central compartment; 𝐴^𝐶𝑅𝑂^ is the amount of ceftriaxone in the

𝑐 𝑝

peripheral compartment; 𝐴^𝐶𝑅𝑂^ is the amount of ceftriaxone in the first transit compartment between the central compartment and the lower intestinal tract; 𝐴^𝐶𝑅𝑂^ is the amount of ceftriaxone in the second transit compartment between the central compartment and the lower intestinal tract; 𝐴^𝐶𝑅𝑂^ is the amount of ceftriaxone in the third transit compartment between the central compartment and the lower intestinal tract; 𝐴^𝐶𝑅𝑂^ is the amount of

𝑓

𝑡1

𝑡2

𝑡3

ceftriaxone in the lower intestinal tract; 𝐴^𝑐ℎ𝑎𝑟𝑐^ is the amount of charcoal in the lower intestinal tract. We denote

𝑓

𝑘^𝐶𝑅𝑂^ the extraintestinal elimination rate from the central compartment; 𝑘^𝐶𝑅𝑂^ and 𝑘^𝐶𝑅𝑂^ the transfer rates

𝑒 21 21

between the central compartment and the peripheral compartment; 𝑘^𝐶𝑅𝑂^ the elimination rate from the central

𝑐𝑡

compartment to the intestinal tract; 𝑘^𝐶𝑅𝑂^ the transfer rate between the intestinal transit compartments; 𝑘^𝐶𝑅𝑂^ the

𝑐𝑡 𝑓

elimination rate from the lower intestinal tract and 𝑘^𝐶𝑅𝑂^ the adsorption rate of ceftriaxone on the charcoal. Initial conditions of these equations are: 𝐴^𝐶𝑅𝑂^ = 0; 𝐴^𝐶𝑅𝑂^ = 0;𝐴^𝐶𝑅𝑂^ = 0;𝐴^𝐶𝑅𝑂^ = 0 𝐴^𝐶𝑅𝑂^ = 0, 𝐴^𝐶𝑅𝑂^ = 0

𝑎𝑑

and 𝐴𝑐ℎ𝑎𝑟𝑐 = 0.

𝑓,0

𝑐,0

𝑝,0

𝑡1,0

𝑡2,0

𝑡3,0

𝑓,0

𝐴𝐶𝑅𝑂

The concentration of total ceftriaxone in the central compartment 𝐶^𝐶𝑅𝑂^ is computed as: 𝐶^𝐶𝑅𝑂^ = ^𝑐^ , where

𝑉^𝐶𝑅𝑂^ is the volume of distribution.

𝑐 𝑐

𝑉𝐶𝑅𝑂

𝐴𝐶𝑅𝑂

The concentration of free ceftriaxone in the lower intestinal tract 𝐶^𝐶𝑅𝑂^ is computed as: 𝐶^𝐶𝑅𝑂^ = ^𝑓^ , where 𝑃

is the fecal weight.

𝑓 𝑓

𝑃𝑓 𝑓

Ceftazidime pharmacokinetic model

The evolution of the amount of ceftazidime in the various compartments was described using the following system of ordinary differential equations:

𝑑𝐴𝐶𝐸𝐹

𝑐

= 𝑘𝐶𝐸𝐹 × 𝐴𝐶𝐸𝐹 − (𝑘𝐶𝐸𝐹 + 𝑘𝐶𝐸𝐹 + 𝑘𝐶𝐸𝐹) × 𝐴𝐶𝐸𝐹

𝑑𝑡

21 𝑝

𝑑𝐴𝐶𝐸𝐹

𝑒 𝑐𝑡 12 𝑐

𝑝 = 𝑘𝐶𝐸𝐹 × 𝐴𝐶𝐸𝐹 − 𝑘𝐶𝐸𝐹 × 𝐴𝐶𝐸𝐹

𝑑𝑡 12 𝑐

𝑑𝐴𝐶𝐸𝐹

21 𝑝

𝑡1 = 𝑘𝐶𝐸𝐹 × 𝐴𝐶𝐸𝐹 − 𝑘𝐶𝐸𝐹 × 𝐴𝐶𝐸𝐹

𝑑𝑡

𝑑𝐴𝐶𝐸𝐹

𝑐𝑡 𝑐 𝑡

𝑡1

𝑡2 = 𝑘𝐶𝐸𝐹 × 𝐴𝐶𝐸𝐹 − 𝑘𝐶𝐸𝐹 × 𝐴𝐶𝐸𝐹

𝑑𝑡 ^𝑡^

𝑑𝐴𝐶𝐸𝐹

𝑡1 𝑡

𝑡2

𝑡3 = 𝑘𝐶𝐸𝐹 × 𝐴𝐶𝐸𝐹 − 𝑘𝐶𝐸𝐹 × 𝐴𝐶𝐸𝐹

𝑑𝐴𝐶𝐸𝐹

𝑑𝑡 ^𝑡^

𝑡2 𝑡

𝑡3

𝑓 = 𝑘𝐶𝐸𝐹 × 𝐴𝐶𝐸𝐹 − 𝑘𝐶𝐸𝐹 × 𝐴𝐶𝐸𝐹 − 𝑘𝐶𝐸𝐹 × 𝐴𝑐ℎ𝑎𝑟𝑐 × 𝐴𝐶𝐸𝐹

𝑑𝑡 ^𝑡^

𝑡3 𝑓

𝑓 𝑎𝑑 𝑓 𝑓

where 𝐴^𝐶𝐸𝐹^ is the amount of ceftazidime in the central compartment; 𝐴^𝐶𝐸𝐹^ is the amount of ceftazidime in the

𝑐 𝑝

peripheral compartment; 𝐴^𝐶𝐸𝐹^ is the amount of ceftazidime in the first transit compartment between the central compartment and the lower intestinal tract; 𝐴^𝐶𝐸𝐹^ is the amount of ceftazidime in the second transit compartment between the central compartment and the lower intestinal tract; 𝐴^𝐶𝐸𝐹^ is the amount of ceftazidime in the third transit compartment between the central compartment and the lower intestinal tract; 𝐴^𝐶𝐸𝐹^ is the amount of

𝑓

𝑡1

𝑡2

𝑡3

ceftazidime in the lower intestinal tract; 𝐴^𝑐ℎ𝑎𝑟𝑐^ is the amount of charcoal in the lower intestinal tract. We denote

𝑓

𝑘^𝐶𝐸𝐹^ the extraintestinal elimination rate from the central compartment; 𝑘^𝐶𝐸𝐹^ and 𝑘^𝐶𝐸𝐹^ the transfer rates

𝑒 21 21

between the central compartment and the peripheral compartment; 𝑘^𝐶𝐸𝐹^ the elimination rate from the central

𝑐𝑡

compartment to the intestinal tract; 𝑘^𝐶𝐸𝐹^ the transfer rate between the intestinal transit compartments; 𝑘^𝐶𝐸𝐹^ the

𝑐𝑡 𝑓

elimination rate from the lower intestinal tract and 𝑘^𝐶𝐸𝐹^ the adsorption rate of ceftriaxone on the charcoal. Initial conditions of these equations are: 𝐴^𝐶𝐸𝐹^ = 0; 𝐴^𝐶𝐸𝐹^ = 0;𝐴^𝐶𝐸𝐹^ = 0;𝐴^𝐶𝐸𝐹^ = 0 𝐴^𝐶𝐸𝐹^ = 0, 𝐴^𝐶𝐸𝐹^ = 0

𝑎𝑑

and 𝐴𝑐ℎ𝑎𝑟𝑐 = 0.

𝑓,0

𝑐,0

𝑝,0

𝑡1,0

𝑡2,0

𝑡3,0

𝑓,0

𝐴𝐶𝐸𝐹

The concentration of total ceftazidime in the central compartment 𝐶^𝐶𝐸𝐹^ is computed as: 𝐶^𝐶𝐸𝐹^ = ^𝑐^ , where

𝑉^𝐶𝐸𝐹^ is the volume of distribution.

𝑐 𝑐

𝑉𝐶𝐸𝐹

𝐴𝐶𝐸𝐹

The concentration of free ceftazidime in the lower intestinal tract 𝐶^𝐶𝐸𝐹^ is computed as: 𝐶^𝐶𝐸𝐹^ = ^𝑓^

, where 𝑃

is the fecal weight.

𝑓 𝑓

𝑃𝑓 𝑓

Avibactam pharmacokinetic model

The evolution of the amount of avibactam in the various compartments was described using the following system of ordinary differential equations:

𝑑𝐴𝐴𝑉𝐼

𝑐 = 𝑘 𝐴𝑉𝐼 × 𝐴𝐴𝑉𝐼 − (𝑘𝐴𝑉𝐼 + 𝑘 𝐴𝑉𝐼 + 𝑘𝐴𝑉𝐼) × 𝐴𝐴𝑉𝐼

𝑑𝑡

21 𝑝

𝑑𝐴𝐴𝑉𝐼

𝑒 𝑐𝑡 12 𝑐

𝑝 = 𝑘 𝐴𝑉𝐼 × 𝐴𝐴𝑉𝐼 − 𝑘 𝐴𝑉𝐼 × 𝐴𝐴𝑉𝐼

𝑑𝑡

12 𝑐

21 𝑝

𝐴𝑉𝐼

𝑑𝐴

𝑡1 = 𝑘 𝐴𝑉𝐼 × 𝐴𝐴𝑉𝐼 − 𝑘 𝐴𝑉𝐼 × 𝐴𝐴𝑉𝐼

𝑑𝑡

𝑑𝐴𝐴𝑉𝐼

𝑐𝑡 𝑐 𝑡

𝑡1

𝑡2 = 𝑘 𝐴𝑉𝐼 × 𝐴𝐴𝑉𝐼 − 𝑘 𝐴𝑉𝐼 × 𝐴𝐴𝑉𝐼

𝑑𝑡 ^𝑡^

𝑑𝐴𝐴𝑉𝐼

𝑡1 𝑡

𝑡2

𝑡3 = 𝑘 𝐴𝑉𝐼 × 𝐴𝐴𝑉𝐼 − 𝑘 𝐴𝑉𝐼 × 𝐴𝐴𝑉𝐼

𝑑𝐴𝐴𝑉𝐼

𝑑𝑡 ^𝑡^

𝑡2 𝑡

𝑡3

𝑓 = 𝑘𝐴𝑉𝐼 × 𝐴𝐴𝑉𝐼 − 𝑘𝐴𝑉𝐼 × 𝐴𝐴𝑉𝐼 − 𝑘 𝐴𝑉𝐼 × 𝐴𝑐ℎ𝑎𝑟𝑐 × 𝐴𝐴𝑉𝐼

𝑑𝑡 ^𝑡^

𝑡3 𝑓

𝑓 𝑎𝑑 𝑓 𝑓

where 𝐴^𝐴𝑉𝐼^ is the amount of avibactam in the central compartment; 𝐴^𝐴𝑉𝐼^ is the amount of avibactam in the

𝑐 𝑝

peripheral compartment; 𝐴^𝐴𝑉𝐼^ is the amount of avibactam in the first transit compartment between the central compartment and the lower intestinal tract; 𝐴^𝐴𝑉𝐼^ is the amount of avibactam in the second transit compartment between the central compartment and the lower intestinal tract; 𝐴^𝐴𝑉𝐼^ is the amount of avibactam in the third transit compartment between the central compartment and the lower intestinal tract; 𝐴^𝐴𝑉𝐼^ is the amount of

𝑓

𝑡1

𝑡2

𝑡3

avibactam in the lower intestinal tract; 𝐴^𝑐ℎ𝑎𝑟𝑐^ is the amount of charcoal in the lower intestinal tract. We denote

𝑓

𝑘^𝐴𝑉𝐼^ the extraintestinal elimination rate from the central compartment; 𝑘^𝐴𝑉𝐼^ and 𝑘^𝐴𝑉𝐼^ the transfer rates

𝑒 21 21

between the central compartment and the peripheral compartment; 𝑘 ^𝐴𝑉𝐼^ the elimination rate from the central

𝑐𝑡

compartment to the intestinal tract; 𝑘 ^𝐴𝑉𝐼^ the transfer rate between the intestinal transit compartments; 𝑘 ^𝐴𝑉𝐼^ the

𝑐𝑡 𝑓

elimination rate from the lower intestinal tract and 𝑘 ^𝐴𝑉𝐼^ the adsorption rate of ceftriaxone on the charcoal. Initial conditions of these equations are: 𝐴^𝐴𝑉𝐼^ = 0; 𝐴^𝐴𝑉𝐼^ = 0;𝐴^𝐴𝑉𝐼^ = 0;𝐴^𝐴𝑉𝐼^ = 0 𝐴^𝐴𝑉𝐼^ = 0, 𝐴^𝐴𝑉𝐼^ = 0 and

𝑎𝑑

𝐴𝑐ℎ𝑎𝑟𝑐 = 0.

𝑓,0

𝑐,0

𝑝,0

𝑡1,0

𝑡2,0

𝑡3,0

𝑓,0

𝐴𝐴𝑉𝐼

The concentration of total avibactam in the central compartment 𝐶^𝐴𝑉𝐼^ is computed as: 𝐶^𝐴𝑉𝐼^ = ^𝑐^ , where

𝑉^𝑎𝑣𝑖^ is the volume of distribution.

𝑐 𝑐

𝑉𝐴𝑉𝐼

𝐴𝐴𝑉𝐼

The concentration of free avibactam in the lower intestinal tract 𝐶^𝐴𝑉𝐼^ is computed as: 𝐶^𝐴𝑉𝐼^ = ^𝑓^

, where 𝑃 is

the fecal weight.

𝑓 𝑓

𝑃𝑓 𝑓

Piperacillin pharmacokinetic model

The evolution of the amount of piperacillin in the various compartments was described using the following system of ordinary differential equations:

𝑑𝐴𝑃𝐼𝑃

𝑐 = 𝑘𝑃𝐼𝑃 × 𝐴𝑃𝐼𝑃 − (𝑘𝑃𝐼𝑃 + 𝑘𝑃𝐼𝑃 + 𝑘𝑃𝐼𝑃) × 𝐴𝑃𝐼𝑃

𝑑𝑡

21 𝑝

𝑑𝐴𝑃𝐼𝑃

𝑒 𝑐𝑡 12 𝑐

𝑝 = 𝑘𝑃𝐼𝑃 × 𝐴𝑃𝐼𝑃 − 𝑘𝑃𝐼𝑃 × 𝐴𝑃𝐼𝑃

𝑑𝑡 12 𝑐

𝑑𝐴𝑃𝐼𝑃

21 𝑝

𝑡1 = 𝑘𝑃𝐼𝑃 × 𝐴𝑃𝐼𝑃 − 𝑘𝑃𝐼𝑃 × 𝐴𝑃𝐼𝑃

𝑑𝑡

𝑑𝐴𝑃𝐼𝑃

𝑐𝑡 𝑐 𝑡

𝑡1

𝑡2 = 𝑘𝑃𝐼𝑃 × 𝐴𝑃𝐼𝑃 − 𝑘𝑃𝐼𝑃 × 𝐴𝑃𝐼𝑃

𝑃𝐼𝑃

𝑑𝐴

𝑡3 = 𝑘𝑃𝐼𝑃 × 𝐴𝑃𝐼𝑃 − 𝑘𝑃𝐼𝑃 × 𝐴𝑃𝐼𝑃

𝑑𝑡 ^𝑡^

𝑑𝐴𝑃𝐼𝑃

𝑡2 𝑡

𝑡3

𝑡4 = 𝑘𝑃𝐼𝑃 × 𝐴𝑃𝐼𝑃 − 𝑘𝑃𝐼𝑃 × 𝐴𝑃𝐼𝑃

𝑑𝐴𝑃𝐼𝑃

𝑑𝑡 ^𝑡^

𝑡3 𝑡

𝑡4

𝑓 = 𝑘𝑃𝐼𝑃 × 𝐴𝑃𝐼𝑃 − 𝑘𝑃𝐼𝑃 × 𝐴𝑃𝐼𝑃 − 𝑘𝑃𝐼𝑃 × 𝐴𝑐ℎ𝑎𝑟𝑐 × 𝐴𝑃𝐼𝑃

𝑑𝑡 ^𝑡^

𝑡4 𝑓

𝑓 𝑎𝑑 𝑓 𝑓

where 𝐴^𝑃𝐼𝑃^ is the amount of piperacillin in the central compartment; 𝐴^𝑃𝐼𝑃^ is the amount of piperacillin in the

𝑐 𝑝

peripheral compartment; 𝐴^𝑃𝐼𝑃^ is the amount of piperacillin in the first transit compartment between the central compartment and the lower intestinal tract; 𝐴^𝑃𝐼𝑃^ is the amount of piperacillin in the second transit compartment between the central compartment and the lower intestinal tract; 𝐴^𝑃𝐼𝑃^ is the amount of piperacillin in the third transit compartment between the central compartment and the lower intestinal tract; 𝐴^𝑃𝐼𝑃^ is the amount of piperacillin in the fourth transit compartment between the central compartment and the lower intestinal tract;

𝑡1

𝑡2

𝑡3

𝑡4

𝐴^𝑃𝐼𝑃^ is the amount of piperacillin in the lower intestinal tract; 𝐴^𝑐ℎ𝑎𝑟𝑐^ is the amount of charcoal in the lower

𝑓 𝑓

intestinal tract. We denote 𝑘^𝑃𝐼𝑃^ the extraintestinal elimination rate from the central compartment; 𝑘^𝑃𝐼𝑃^ and

𝑒 21

𝑘^𝑃𝐼𝑃^ the transfer rates between the central compartment and the peripheral compartment; 𝑘^𝑃𝐼𝑃^ the elimination

21 𝑐𝑡

rate from the central compartment to the intestinal tract; 𝑘^𝑃𝐼𝑃^ the transfer rate between the intestinal transit

𝑐𝑡

compartments; 𝑘^𝑃𝐼𝑃^ the elimination rate from the lower intestinal tract and 𝑘^𝑃𝐼𝑃^ the adsorption rate of

𝑓

ceftriaxone on the charcoal.

𝑎𝑑

Initial conditions of these equations are: 𝐴^𝑃𝐼𝑃^ = 0; 𝐴𝑝,0 = 0;𝐴^𝑃𝐼𝑃^ = 0;𝐴^𝑃𝐼𝑃^ = 0 𝐴^𝑃𝐼𝑃^ = 0; 𝐴^𝑃𝐼𝑃^ = 0,

𝐴𝑃𝐼𝑃 = 0 and 𝐴𝑐ℎ𝑎𝑟𝑐 = 0.

𝑐,0

𝑡1,0

𝑡2,0

𝑡3,0

𝑡4,0

𝑓,0

𝑓,0

𝐴𝑃𝐼𝑃

The concentration of total piperacillin in the central compartment 𝐶^𝑃𝐼𝑃^ is computed as: 𝐶^𝑃𝐼𝑃^ = ^𝑐^ , where

𝑉^𝑃𝐼𝑃^ is the volume of distribution.

𝑐 𝑐

𝑉𝑃𝐼𝑃

𝐴𝑃𝐼𝑃

The concentration of free piperacillin in the lower intestinal tract 𝐶^𝑃𝐼𝑃^ is computed as: 𝐶^𝑃𝐼𝑃^ = ^𝑓^ , where 𝑃

is the fecal weight.

𝑓 𝑓

𝑃𝑓 𝑓

Tazobactam pharmacokinetic model

The evolution of the amount of tazobactam in the various compartments was described using the following system of ordinary differential equations:

𝑑𝐴𝑇𝑍𝐵

𝑐 = 𝑘𝑇𝑍𝐵 × 𝐴𝑇𝑍𝐵 − (𝑘𝑇𝑍𝐵 + 𝑘𝑇𝑍𝐵 + 𝑘𝑇𝑍𝐵) × 𝐴𝑇𝑍𝐵

𝑑𝑡

21 𝑝

𝑑𝐴𝑇𝑍𝐵

𝑒 𝑐𝑡 12 𝑐

𝑝 = 𝑘𝑇𝑍𝐵 × 𝐴𝑇𝑍𝐵 − 𝑘𝑇𝑍𝐵 × 𝐴𝑇𝑍𝐵

𝑑𝑡 12 𝑐

𝑑𝐴𝑇𝑍𝐵

21 𝑝

𝑡1 = 𝑘𝑇𝑍𝐵 × 𝐴𝑇𝑍𝐵 − 𝑘𝑇𝑍𝐵 × 𝐴𝑇𝑍𝐵

𝑑𝑡

𝑑𝐴𝑇𝑍𝐵

𝑐𝑡 𝑐 𝑡

𝑡1

𝑡2 = 𝑘𝑇𝑍𝐵 × 𝐴𝑇𝑍𝐵 − 𝑘𝑇𝑍𝐵 × 𝐴𝑇𝑍𝐵

𝑇𝑍𝐵

𝑑𝐴

𝑡3 = 𝑘𝑇𝑍𝐵 × 𝐴𝑇𝑍𝐵 − 𝑘𝑇𝑍𝐵 × 𝐴𝑇𝑍𝐵

𝑑𝑡 ^𝑡^

𝑑𝐴𝑇𝑍𝐵

𝑡2 𝑡

𝑡3

𝑡4 = 𝑘𝑇𝑍𝐵 × 𝐴𝑇𝑍𝐵 − 𝑘𝑇𝑍𝐵 × 𝐴𝑇𝑍𝐵

𝑑𝐴𝑇𝑍𝐵

𝑑𝑡 ^𝑡^

𝑡3 𝑡

𝑡4

𝑓 = 𝑘𝑇𝑍𝐵 × 𝐴𝑇𝑍𝐵 − 𝑘𝑇𝑍𝐵 × 𝐴𝑇𝑍𝐵 − 𝑘𝑇𝑍𝐵 × 𝐴𝑐ℎ𝑎𝑟𝑐 × 𝐴𝑇𝑍𝐵

𝑑𝑡 ^𝑡^

𝑡4 𝑓

𝑓 𝑎𝑑 𝑓 𝑓

where 𝐴^𝑇𝑍𝐵^ is the amount of tazobactam in the central compartment; 𝐴^𝑇𝑍𝐵^ is the amount of tazobactam in the

𝑐 𝑝

peripheral compartment; 𝐴^𝑇𝑍𝐵^ is the amount of tazobactam in the first transit compartment between the central compartment and the lower intestinal tract; 𝐴^𝑇𝑍𝐵^ is the amount of tazobactam in the second transit compartment between the central compartment and the lower intestinal tract; 𝐴^𝑇𝑍𝐵^ is the amount of tazobactam in the third transit compartment between the central compartment and the lower intestinal tract; 𝐴^𝑇𝑍𝐵^ is the amount of tazobactam in the fourth transit compartment between the central compartment and the lower intestinal tract;

𝑡1

𝑡2

𝑡3

𝑡4

𝐴^𝑇𝑍𝐵^ is the amount of tazobactam in the lower intestinal tract; 𝐴^𝑐ℎ𝑎𝑟𝑐^ is the amount of charcoal in the lower

𝑓 𝑓

intestinal tract. We denote 𝑘^𝑇𝑍𝐵^ the extraintestinal elimination rate from the central compartment; 𝑘^𝑇𝑍𝐵^ and

𝑒 21

𝑘^𝑇𝑍𝐵^ the transfer rates between the central compartment and the peripheral compartment; 𝑘^𝑇𝑍𝐵^ the elimination

21 𝑐𝑡

rate from the central compartment to the intestinal tract; 𝑘^𝑇𝑍𝐵^ the transfer rate between the intestinal transit

𝑐𝑡

compartments; 𝑘^𝑇𝑍𝐵^ the elimination rate from the lower intestinal tract and 𝑘^𝑇𝑍𝐵^ the adsorption rate of

𝑓

ceftriaxone on the charcoal.

𝑎𝑑

Initial conditions of these equations are: 𝐴^𝑇𝑍𝐵^ = 0; 𝐴^𝑇𝑍𝐵^ = 0;𝐴^𝑇𝑍𝐵^ = 0;𝐴^𝑇𝑍𝐵^ = 0 𝐴^𝑇𝑍𝐵^ = 0; 𝐴^𝑇𝑍𝐵^ = 0,

𝐴𝑇𝑍𝐵 = 0 and 𝐴𝑐ℎ𝑎𝑟𝑐 = 0.

𝑐,0

𝑝,0

𝑡1,0

𝑡2,0

𝑡3,0

𝑡4,0

𝑓,0

𝑓,0

𝐴𝑇𝑍𝐵

The concentration of total tazobactam in the central compartment 𝐶^𝑇𝑍𝐵^ is computed as: 𝐶^𝑇𝑍𝐵^ = ^𝑐^ , where

𝑉^𝑇𝑍𝐵^ is the volume of distribution.

𝑐 𝑐

𝑉𝑇𝑍𝐵

𝐴𝑇𝑍𝐵

The concentration of free tazobactam in the lower intestinal tract 𝐶^𝑇𝑍𝐵^ is computed as: 𝐶^𝑇𝑍𝐵^ = ^𝑓^ , where 𝑃

is the fecal weight.

𝑓 𝑓

𝑃𝑓 𝑓

# Supplementary Table S1. Parameter estimates of the final pharmacokinetic model of ceftriaxone.

𝑉 is the volume of distribution; 𝑘𝑒 is the extraintestinal elimination rate from the central compartment; 𝑘_12_ and

𝑘_21_ are the transfer rates between the central compartment and the peripheral compartment; 𝑘𝑐𝑡 is the elimination rate from the central compartment to the intestinal tract; 𝑘𝑡 is the transfer rate between the intestinal transit compartments; 𝑘𝑓 is the elimination rate from the lower intestinal tract; 𝑘^𝐷𝐴𝑉132^ is the transit delivery rate constant of DAV132; 𝑘^𝑐ℎ𝑎𝑟𝑐^ is the elimination rate constant of activated charcoal; 𝑘𝑎𝑑 is the adsorption rate

𝑡

𝑡

constant of ceftriaxone on the active charcoal; 𝜎𝑠𝑙𝑜𝑝𝑒,𝑐 and 𝜎𝑠𝑙𝑜𝑝𝑒,𝑓 are the proportional components of the

residual error for the plasma and fecal pharmacokinetic model, respectively. CRO, ceftriaxone.

|  | **Fixed effects (r.s.e. %)** | **Standard deviation of the exponential**  **random effects (r.s.e. %)** |
| --- | --- | --- |
| **Ceftriaxone** |  |  |
| *Plasma* |  |  |
| 𝑉^𝐶𝑅𝑂^(L) | 4.69 (2.4) | 0.12 (15.7) |
| 𝑘^𝐶𝑅𝑂^ (day^-1^)  𝑒 | 0.17 (2.3) | 0.11 (15.7) |
| 𝑘^𝐶𝑅𝑂^ (day^-1^)  12 | 0.53 (4.4) | 0.12 (37.8) |
| 𝑘^𝐶𝑅𝑂^ (day^-1^)  21 | 0.47 (3.8) | 0.12 (54.3) |
| *Feces* |  |  |
| 𝑘^𝐶𝑅𝑂^ (day^-1^)  𝑐𝑡 | 0.00030 (50.1) | 2.73 (13.5) |
| 𝑘^𝐶𝑅𝑂^ (day^-1^)  𝑡 | 0.14 (17.6) | 0.85 (21.7) |
| 𝑘^𝐶𝑅𝑂^ (day^-1^)  𝑓 | 0.10 (18.1) | 0.35 (50.7) |
| **DAV132** |  |  |
| 𝑘𝐷𝐴𝑉132 (day-1)  𝑡 | 0.102 (fixed) | 0.34 (fixed) |
| 𝑘𝑐ℎ𝑎𝑟𝑐 (day-1)  𝑡 | 0.046 (fixed) | 1.42 (fixed) |
| **Drug effect** |  |  |
| 𝑘^𝐶𝑅𝑂^ (day^-1^)  𝑎𝑑 | 0.000044 (54.8) | 1.87 (27.9) |
| **Residual error** |  |  |
| 𝜎𝐶𝑅𝑂  𝑠𝑙𝑜𝑝𝑒,𝑐 | 0.14 (2.7) |  |
| 𝜎𝐶𝑅𝑂  𝑠𝑙𝑜𝑝𝑒,𝑓 | 0.61 (5.8) |  |

# Supplementary Table S2. Parameter estimates of the final pharmacokinetic model of ceftazidime and avibactam.

𝑉 is the volume of distribution; 𝑘𝑒 is the extraintestinal elimination rate from the central compartment; 𝑘_12_ and

𝑘_21_ are the transfer rates between the central compartment and the peripheral compartment; 𝑘𝑐𝑡 is the elimination rate from the central compartment to the intestinal tract; 𝑘𝑡 is the transfer rate between the intestinal transit compartments; 𝑘𝑓 is the elimination rate from the lower intestinal tract; 𝑘^𝐷𝐴𝑉132^ is the transit delivery rate constant of DAV132; 𝑘^𝑐ℎ𝑎𝑟𝑐^ is the elimination rate constant of activated charcoal; 𝑘𝑎𝑑 is the adsorption rate

𝑡

𝑡

constant of ceftriaxone on the active charcoal; 𝜌(𝑘^𝐶𝐸𝐹^, 𝑘^𝐴𝑉𝐼^) is the correlation coefficient between the

𝑒 𝑒

extraintestinal elimination rate from the central compartment of ceftazidime and of avibactam; 𝜎𝑖𝑛𝑡𝑒𝑟,𝑐 and

𝜎𝑠𝑙𝑜𝑝𝑒,𝑐 are the additive and proportional components of the residual error for the plasma pharmacokinetic model, respectively; 𝜎𝑠𝑙𝑜𝑝𝑒,𝑓 is the proportional components of the residual error for the fecal pharmacokinetic model. AVI, avibactam; CEF, ceftazidime.

|  | **Fixed effects (r.s.e. %)** | **Standard deviation of the exponential**  **random effects (r.s.e. %)** |
| --- | --- | --- |
| **Ceftazidime** |  |  |
| *Plasma* |  |  |
| 𝑉^𝐶𝐸𝐹^(L) | 9.67 (3.1) | 0.17 (12.9) |
| 𝑘^𝐶𝐸𝐹^ (day^-1^)  𝑒 | 0.64 (2.5) | 0.13 (15) |
| 𝑘^𝐶𝐸𝐹^ (day^-1^)  12 | 0.61 (3.7) | 0.072 (53) |
| 𝑘^𝐶𝐸𝐹^ (day^-1^)  21 | 0.83 (3.2) | 0.11 (22.5) |
| *Feces* |  |  |
| 𝑘^𝐶𝐸𝐹^ (day^-1^)  𝑐𝑡 | 0.0017 (42.4) | 1.79 (13.9) |
| 𝑘^𝐶𝐸𝐹^ (day^-1^)  𝑡 | 0.24 (28.3) | 1.44 (15.7) |
| 𝑘^𝐶𝐸𝐹^ (day^-1^)  𝑓 | 0.086 (23) | 0.60 (27.9) |
| **Avibactam** |  |  |
| *Plasma* |  |  |
| 𝑉 ^𝐴𝑉𝐼^(L) | 11.28 (5.2) | 0.30 (27.7) |
| 𝑘^𝐴𝑉𝐼^ (day^-1^)  𝑒 | 1.00 (3.5) | 0.20 (15.8) |
| 𝑘 ^𝐴𝑉𝐼^ (day^-1^)  12 | 0.46 (0) | 0 (fixed) |
| 𝑘 ^𝐴𝑉𝐼^ (day^-1^)  21 | 0.73 (2.8) | 0.13 (18.7) |
| *Feces* |  |  |
| 𝑘 ^𝐴𝑉𝐼^ (day^-1^)  𝑐𝑡 | 0.00054 (30.1) | 1.62 (13.4) |
| 𝑘 ^𝐴𝑉𝐼^ (day^-1^)  𝑡 | 0.38 (42) | 1.97 (18.3) |
| 𝑘 ^𝐴𝑉𝐼^ (day^-1^)  𝑓 | 0.083 (14.9) | 0.61 (47.2) |

|  | **Fixed effects (r.s.e. %)** | **Standard deviation of the exponential**  **random effects (r.s.e. %)** |
| --- | --- | --- |
| **DAV132** |  |  |
| 𝑘𝐷𝐴𝑉132 (day-1)  𝑡 | 0.102 (fixed) | 0.34 (fixed) |
| 𝑘𝑐ℎ𝑎𝑟𝑐 (day-1)  𝑡 | 0.046 (fixed) | 1.42 (fixed) |
| **Drug effect** |  |  |
| 𝑘^𝐶𝐸𝐹^ (day^-1^)  𝑎𝑑 | 0.00019 (67.5) | 2.09 (28) |
| 𝑘 ^𝐴𝑉𝐼^ (day^-1^)  𝑎𝑑 | 0 (fixed) | 0 (fixed) |
| **Correlation** |  |  |
| 𝜌(𝑘𝐶𝐸𝐹, 𝑘𝐴𝑉𝐼)  𝑒 𝑒 | 0.80 (15.9) |  |
| **Residual error** |  |  |
| 𝜎𝐶𝐸𝐹  𝑖𝑛𝑡𝑒𝑟,𝑐 | 0.027 (20.7) |  |
| 𝜎𝐶𝐸𝐹  𝑠𝑙𝑜𝑝𝑒,𝑐 | 0.13 (3.2) |  |
| 𝜎𝐶𝐸𝐹  𝑠𝑙𝑜𝑝𝑒,𝑓 | 0.84 (7.2) |  |
| 𝜎𝐴𝑉𝐼  𝑖𝑛𝑡𝑒𝑟,𝑐 | 0.025 (15.4) |  |
| 𝜎𝐴𝑉𝐼  𝑠𝑙𝑜𝑝𝑒,𝑐 | 0.13 (7.9) |  |
| 𝜎𝐴𝑉𝐼  𝑠𝑙𝑜𝑝𝑒,𝑓 | 0.68 (6.5) |  |

# Supplementary Table S3. Parameter estimates of the final pharmacokinetic model of piperacillin and tazobactam.

𝑉 is the volume of distribution; 𝑘𝑒 is the extraintestinal elimination rate from the central compartment; 𝑘_12_ and

𝑘_21_ are the transfer rates between the central compartment and the peripheral compartment; 𝑘𝑐𝑡 is the elimination rate from the central compartment to the intestinal tract; 𝑘𝑡 is the transfer rate between the intestinal transit compartments; 𝑘𝑓 is the elimination rate from the lower intestinal tract; 𝑘^𝐷𝐴𝑉132^ is the transit delivery rate constant of DAV132; 𝑘^𝑐ℎ𝑎𝑟𝑐^ is the elimination rate constant of activated charcoal; 𝑘𝑎𝑑 is the adsorption rate

𝑡

𝑡

constant of ceftriaxone on the active charcoal; 𝜎𝑖𝑛𝑡𝑒𝑟,𝑐 and 𝜎𝑠𝑙𝑜𝑝𝑒,𝑐 are the additive and proportional components

of the residual error for the plasma pharmacokinetic model, respectively; 𝜎𝑠𝑙𝑜𝑝𝑒,𝑓 is the proportional components of the residual error for the fecal pharmacokinetic model. PIP, piperacillin; TZB, tazobactam.

|  | **Fixed effects (r.s.e. %)** | **Standard deviation of the exponential**  **random effects (r.s.e. %)** |
| --- | --- | --- |
| **Piperacilin** |  |  |
| *Plasma* |  |  |
| 𝑉^𝑃𝐼𝑃^(L) | 8.88 (3.4) | 0 (fixed) |
| 𝑘^𝑃𝐼𝑃^ (day^-1^)  𝑒 | 1.28 (5.4) | 0.26 (13) |
| 𝑘^𝑃𝐼𝑃^ (day^-1^)  12 | 0.69 (19.2) | 0.42 (23.8) |
| 𝑘^𝑃𝐼𝑃^ (day^-1^)  21 | 1.36 (12.7) | 0.56 (17.1) |
| *Feces* |  |  |
| 𝑘^𝑃𝐼𝑃^ (day^-1^)  𝑐𝑡 | 0.01 (123.6) | 1.82 (62.7) |
| 𝑘^𝑃𝐼𝑃^ (day^-1^)  𝑡 | 0.29 (29.6) | 1.08 (22) |
| 𝑘^𝑃𝐼𝑃^ (day^-1^)  𝑓 | 0.65 (144) | 1.83 (54.8) |
| **Tazobactam** |  |  |
| *Plasma* |  |  |
| 𝑉^𝑇𝑍𝐵^(L) | 9.02 (3.2) | 0 (fixed) |
| 𝑘^𝑇𝑍𝐵^ (day^-1^)  𝑒 | 1.19 (4.9) | 0.24 (13) |
| 𝑘^𝑇𝑍𝐵^ (day^-1^)  12 | 0.82 (13) | 0.38 (23.1) |
| 𝑘^𝑇𝑍𝐵^ (day^-1^)  21 | 1.53 (8.4) | 0.40 (19.7) |
| *Feces* |  |  |
| 𝑘^𝑇𝑍𝐵^ (day^-1^)  𝑐𝑡 | 0.0043 (44.1) | 1.19 (37.4) |
| 𝑘^𝑇𝑍𝐵^ (day^-1^)  𝑡 | 0.43 (26.2) | 1.35 (15.7) |
| 𝑘^𝑇𝑍𝐵^ (day^-1^)  𝑓 | 0.41 (46.7) | 1.47 (31.3) |
| **DAV132** |  |  |
| 𝑘𝐷𝐴𝑉132 (day-1)  𝑡 | 0.102 (fixed) | 0.34 (fixed) |

| 𝑘𝑐ℎ𝑎𝑟𝑐 (day-1)  𝑡 | 0.046 (fixed) | 1.42 (fixed) |
| --- | --- | --- |
| **Drug effect** |  |  |
| 𝑘^𝑃𝐼𝑃^ (day^-1^)  𝑎𝑑 | 0.32 (246.4) | 8.75 (25.4) |
| 𝑘^𝑇𝑍𝐵^ (day^-1^)  𝑎𝑑 | 0.00032 (81.9) | 2.81 (29) |
| **Residual error** |  |  |
| 𝜎𝑃𝐼𝑃  𝑖𝑛𝑡𝑒𝑟,𝑐 | 0.10 (14.5) |  |
| 𝜎𝑃𝐼𝑃  𝑠𝑙𝑜𝑝𝑒,𝑐 | 0.24 (3.6) |  |
| 𝜎𝑃𝐼𝑃  𝑠𝑙𝑜𝑝𝑒,𝑓 | 0.74 (6.8) |  |
| 𝜎𝑇𝑍𝐵  𝑠𝑙𝑜𝑝𝑒,𝑐 | 0.25 (2.9) |  |
| 𝜎𝑇𝑍𝐵  𝑠𝑙𝑜𝑝𝑒,𝑓 | 0.70 (7) |  |

# Supplementary Table S4. Parameter estimates of the final pharmacodynamic model of the effect of ceftriaxone, ceftazidime and piperacillin on the Shannon index.

𝑆𝐼0 is the value of the Shannon index at steady state; 𝑘𝑜𝑢𝑡,𝑆 the first-order elimination rate of the Shannon index from the lower intestinal tract; 𝐸𝑚𝑎𝑥 is the maximal effect of the antibiotic on the elimination rate of the Shannon index; 𝐸𝐶50 is the concentration of moxifloxacin leading to 50% of the maximal effect, 𝜎𝑖𝑛𝑡𝑒𝑟,𝑆 is the additive component of the residual error for the pharmacodynamic model of the Shannon index. CRO, ceftriaxone; CEF, ceftazidime; PIP, piperacillin

|  | **Fixed effects (r.s.e. %)** | **Standard deviation of the exponential**  **random effects (r.s.e. %)** |
| --- | --- | --- |
| 𝑆𝐼_0_ (Shannon unit) | 4.70 (1.2) | 0.11 (8.4) |
| 𝑘𝑜𝑢𝑡,𝑆 (day^-1^) | 0.0096 (10.5) | 0.39 (27.9) |
| **Ceftriaxone** |  |  |
| 𝐸𝐶𝑅𝑂  𝑚𝑎𝑥 | 0.071 (39.1) | 1.20 (21.9) |
| 𝐸𝐶^𝐶𝑅𝑂^ (µg/g)  50 | 0.15 (18.9) | 0 (fixed) |
| **Ceftazidime** |  |  |
| 𝐸𝐶𝐸𝐹  𝑚𝑎𝑥 | 0.58 (22.4) | 1.01 (21.1) |
| 𝐸𝐶^𝐶𝐸𝐹^ (µg/g)  50 | 10.90 (23.7) | 0 (fixed) |
| **Piperacillin** |  |  |
| 𝐸𝑃𝐼𝑃  𝑚𝑎𝑥 | 0.78 (1) | 0 (fixed) |
| 𝐸𝐶^𝑃𝐼𝑃^ (µg/g)  50 | 6.68 (122.8) | 5.59 (20.1) |
| **Residual error** |  |  |
| 𝜎𝑖𝑛𝑡𝑒𝑟,𝑆 (Shannon u | nit) 0.55 (2.8) |  |

# Supplementary Table S5. Derived pharmacokinetic and pharmacodynamic indices of the impact of antibiotics on the bacterial gut microbiota, according to various treatment durations.

In order to gain insight into the effect of antibiotics, we computed two pharmacodynamic indices relative to baseline values, the maximal loss of the Shannon diversity index (expressed in percentage of the baseline value) and the AUC of the change from baseline of the Shannon diversity index. In the absence of antibiotic treatment, values for these indices are 0. Results were obtained for each antibiotic from 1000 individuals simulated in the asymptotic distribution of the parameters estimated in the final model. Data are presented as medians along with their 90% prediction intervals. AUC, area under the curve; CRO, Ceftriaxone; CZA, Ceftazidime/avibactam; TZP, Piperacillin/tazobactam; MXF, Moxifloxacin.

| **Simulated treatment duration** | | | | | |
| --- | --- | --- | --- | --- | --- |
|  | **3 days** | **5 days** | **7 days** | **10 days** | **14 days** |
| **Maximal fecal concentration (µg/g)** | | | | | |
| CRO | 3.1 [0.5; 20.1] | 3.5 [0.6; 23.4] | 3.7 [0.6; 24] | 3.7 [0.6; 24.5] | 3.7 [0.6; 24.5] |
| CZA | 33.6 [10.7; 117.5] | 35.9 [11.3; 124.8] | 36.4 [11.6; 125.3] | 36.7 [11.8; 127.2] | 37 [11.8; 127.2] |
| TZP | 25.2 [4.3; 128.9] | 26 [4.6; 132.5] | 26.3 [4.7; 136.4] | 26.4 [4.7; 140.5] | 26.4 [4.7; 141.4] |
| MXF | 72.6 [57.5; 95.1] | 80.7 [62; 109.2] | 82.4 [62.8; 112.9] | 83 [62.9; 114.1] | 83.1 [63.1; 114.8] |
| **Time to decrease below the lower limit of quantification after the beginning of the last dose (days)** | | | | | |
| CRO | 5.1 [3.5; 6.9] | 5.1 [3.5; 6.9] | 5.1 [3.5; 6.9] | 5.1 [3.5; 6.8] | 5 [3.5; 6.6] |
| CZA | 4.9 [3.3; 7.8] | 4.9 [3.2; 7.6] | 4.8 [3.2; 7.5] | 4.7 [3.2; 7.3] | 4.4 [3; 6.8] |
| TZP | 2.5 [1.4; 5.1] | 2.5 [1.4; 5] | 2.5 [1.4; 5] | 2.4 [1.3; 4.9] | 2.3 [1.3; 4.5] |
| MXF | 11.8 [10.4; 13.3] | 12.2 [10.8; 13.6] | 12.3 [10.9; 13.7] | 12.2 [10.8; 13.4] | 10.8 [10; 11.4] |
| **Time to maximal loss (days)** | | | | | |
| CRO | 5.9 [4.9; 7.1] | 7.4 [6.5; 8.7] | 9.1 [8.1; 10.0] | 11.6 [11.0; 13.0] | 15.0 [14.0; 16.0] |
| CZA | 4.4 [3.8; 5.5] | 6.0 [5.4; 7.1] | 7.7 [7.3; 8.5] | 10.4 [10.0; 11.0] | 14.2 [14.0; 15.0] |
| TZP | 4.1 [3.4; 5.7] | 5.8 [5.3; 7.2] | 7.7 [7.2; 8.8] | 10.4 [10.0; 11.0] | 14.2 [14.0; 15.0] |
| MXF | 5.0 [4.2; 5.8] | 6.4 [5.8; 7.3] | 8.0 [7.4; 8.9] | 10.6 [10.0; 11.0] | 14.2 [14.0; 15.0] |
| **Maximal loss (Shannon unit)** | | | | | |
| CRO | 0.2 [0.1; 0.4] | 0.2 [0.1; 0.4] | 0.2 [0.1; 0.5] | 0.2 [0.1; 0.5] | 0.2 [0.1; 0.51] |
| CZA | 0.8 [0.4; 1.5] | 1.0 [0.5; 1.8] | 1.1 [0.5; 1.9] | 1.2 [0.6; 2.0] | 1.3 [0.7; 2.0] |
| TZP | 1.0 [0.1; 1.7] | 1.2 [0.9; 1.9] | 1.4 [0.1; 2.0] | 1.5 [0.1; 2.0] | 1.5 [0.1; 2.0] |
| MXF | 0.8 [0.4; 1.3] | 1.0 [0.6; 1.6] | 1.2 [0.7; 1.7] | 1.3 [0.9; 1.8] | 1.4 [0.9; 1.9] |

| **Simulated treatment duration** | | | | | |
| --- | --- | --- | --- | --- | --- |
|  | **3 days** | **5 days** | **7 days** | **10 days** | **14 days** |
| **Maximal loss (% of baseline value)** | | | | | |
| CRO | 3.4 [1.4; 8.1] | 4.0 [1.7; 9.1] | 4.3 [1.8; 9.9] | 4.7 [02.0; 11.0] | 5.0 [2.2; 11.0] |
| CZA | 17.7 [7.8; 33.0] | 21.7 [10.0; 37.0] | 24.0 [12.0; 40.0] | 25.5 [13.0; 42.0] | 26.6 [14.0; 43.0] |
| TZP | 21.5 [1.3; 36.0] | 27.3 [1.9; 40.0] | 30.1 [2.2; 42.0] | 32.0 [2.4; 43.0] | 33.0 [2.5; 44.0] |
| MXF | 17.2 [9.4; 28.0] | 21.9 [13.0; 34.0] | 24.8 [15.0; 37.0] | 27.9 [18.0; 39.0] | 30.7 [21.0; 41.0] |
| **Time to return to 95% of baseline value after the last administration (days)** | | | | | |
| CRO | 4.8 [3.3; 8.3] | 4.8 [2.9; 8.6] | 4.9 [2.6; 8.7] | 4.8 [2.3; 8.9] | 4.4 [1.9; 8.9] |
| CZA | 8.6 [4.8; 12.3] | 9.3 [5.8; 12.8] | 9.4 [6; 13.3] | 9.7 [6.4; 13.3] | 9.9 [6.5; 13.4] |
| TZP | 7.5 [1.5; 14.2] | 8.3 [1.4; 14.7] | 8.5 [1.2; 15.1] | 8.8 [1; 15.6] | 8.9 [0.8; 15.7] |
| MXF | 11.5 [6.7; 17.1] | 13.2 [8.3; 19.1] | 14.2 [9; 20.6] | 14.9 [9.7; 22.3] | 15.4 [9.9; 23.5] |
| **AUC between day 0 and day 42 of the change from baseline of the Shannon diversity index (Shannon unit.day)** | | | | | |
| CRO | -1.6 [-3.9; -0.6] | -2.1 [-5.0; -0.8] | -2.6 [-6.3; -1.1] | -3.3 [-7.5; -1.4] | -4.4 [-9.6; -1.8] |
| CZA | -7.0 [-13.8; -3.0] | -10.1 [-18.3; -4.6] | -13.0 [-22.5; -5.8] | -17.0 [-28.8; -7.9] | -22.1 [-36.7; -10.8] |
| TZP | -7.3 [-15.8; -0.4] | -10.9 [-20.4; -0.6] | -14.3 [-25.0; -0.9] | -19.2 [-31.3; -1.3] | -25.8 [-39.6; -1.8] |
| MXF | -9.3 [-15.0; -5.4] | -13.2 [-20.4; -7.9] | -16.7 [-25.0; -10.0] | -21.6 [-31.7; -13.3] | -27.9 [-39.6; -17.9] |

# Supplementary Figure S1

Spaghetti plot of the observed concentrations of ceftriaxone in plasma (top) and feces (bottom) for the 36 subjects treated with ceftriaxone.

NODAV refers to subjects treated with ceftriaxone only (N=12); DAVD1 refers to subjects treated with ceftriaxone and the low dose of DAV132 (N=12); DAVD2 refers to subjects treated with ceftriaxone and the high dose of DAV132 (N=12). CRO, ceftriaxone.


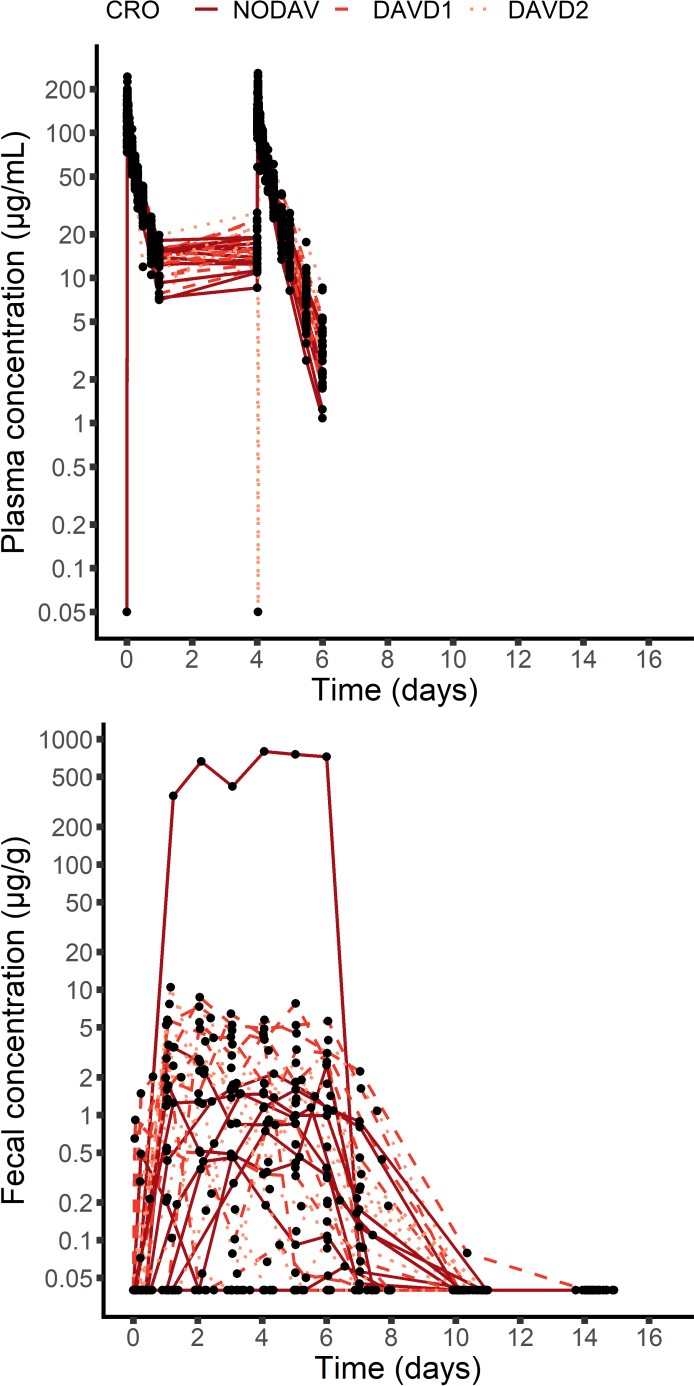


# Supplementary Figure S2

Spaghetti plot of the observed concentrations of ceftazidime (left, blue) and avibactam (right, purple) in plasma (top) and feces (bottom) for the 36 subjects treated with ceftazidime/avibactam.

NODAV refers to subjects treated with ceftazidime/avibactam only (N=12); DAVD1 refers to subjects treated with ceftazidime/avibactam and the low dose of DAV132 (N=12); DAVD2 refers to subjects treated with ceftazidime/avibactam and the high dose of DAV132 (N=12). CEF, ceftazidime; AVI, avibactam.


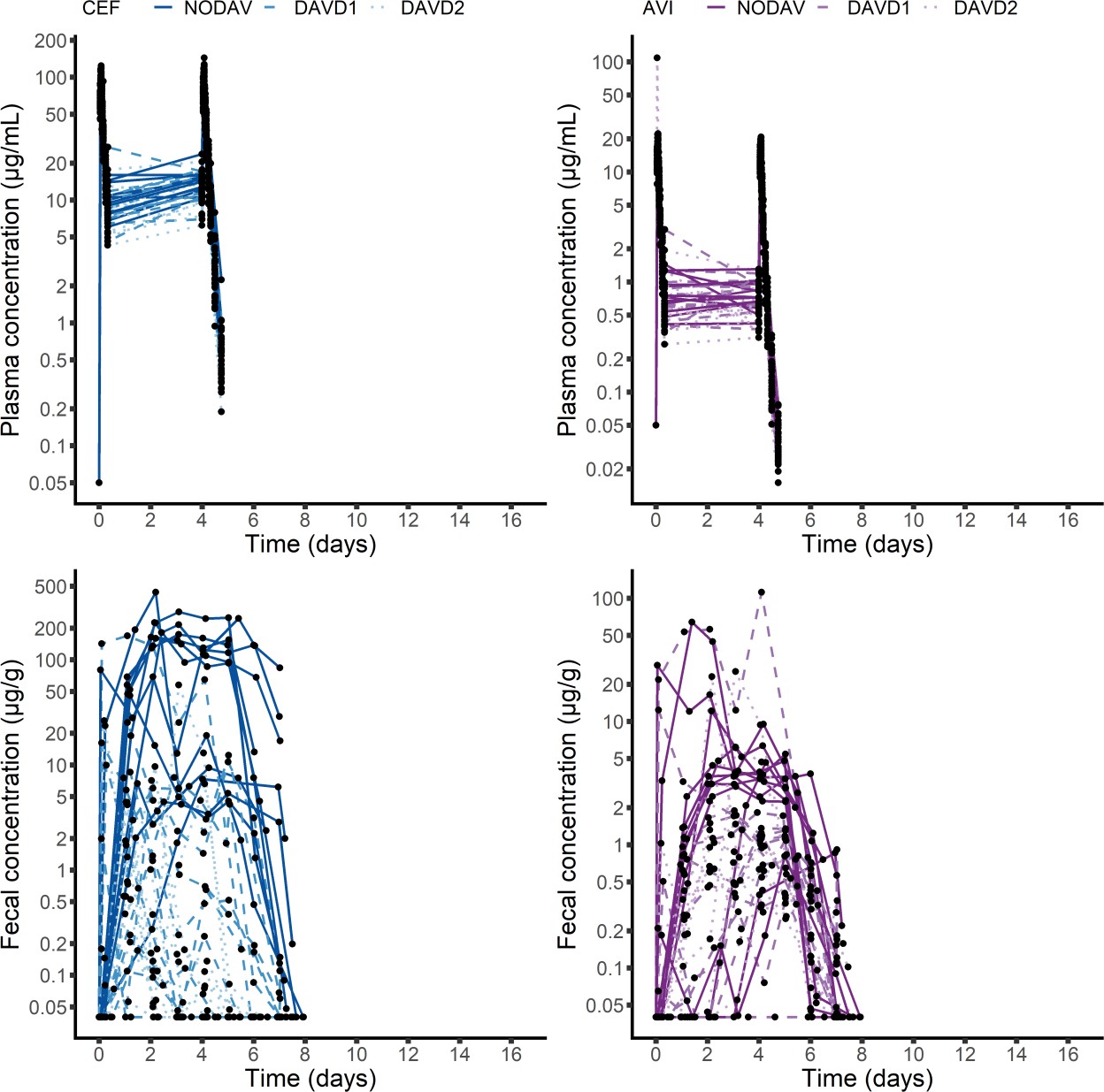


# Supplementary Figure S3

Spaghetti plot of the observed concentrations of piperacillin (left, green) and tazobactam (right, brown) in plasma (top) and feces (bottom) for the 37 subjects treated with piperacillin/tazobactam.

NODAV refers to subjects treated with piperacillin/tazobactam only (N=12); DAVD1 refers to subjects treated with piperacillin/tazobactam and the low dose of DAV132 (N=12); DAVD2 refers to subjects treated with piperacillin/tazobactam and the high dose of DAV132 (N=13). PIP, piperacillin; TAZ, tazobactam.


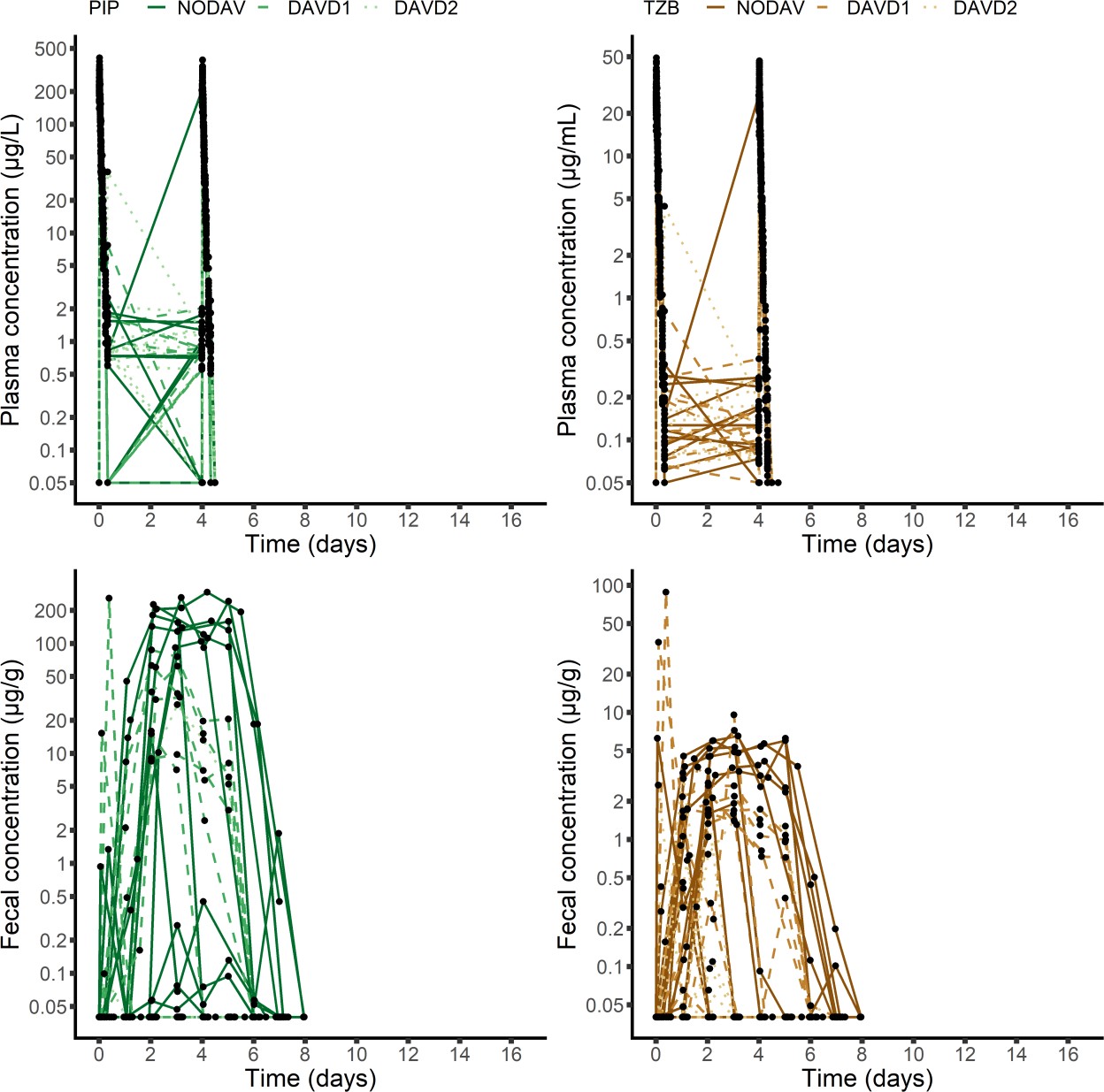


# Supplementary Figure S4

Individual fits for the plasma ceftriaxone concentrations for the final pharmacokinetic model in the 36 subjects treated with ceftriaxone. Black dots represent observed ceftriaxone concentrations. Red curves represent the individual pharmacokinetic profiles predicted by the model using estimated individual parameters. NODAV refers to subjects treated with ceftriaxone only (N=12); DAVD1 refers to subjects treated with ceftriaxone and the low dose of DAV132 (N=12); DAVD2 refers to subjects treated with ceftriaxone and the high dose of DAV132 (N=12).


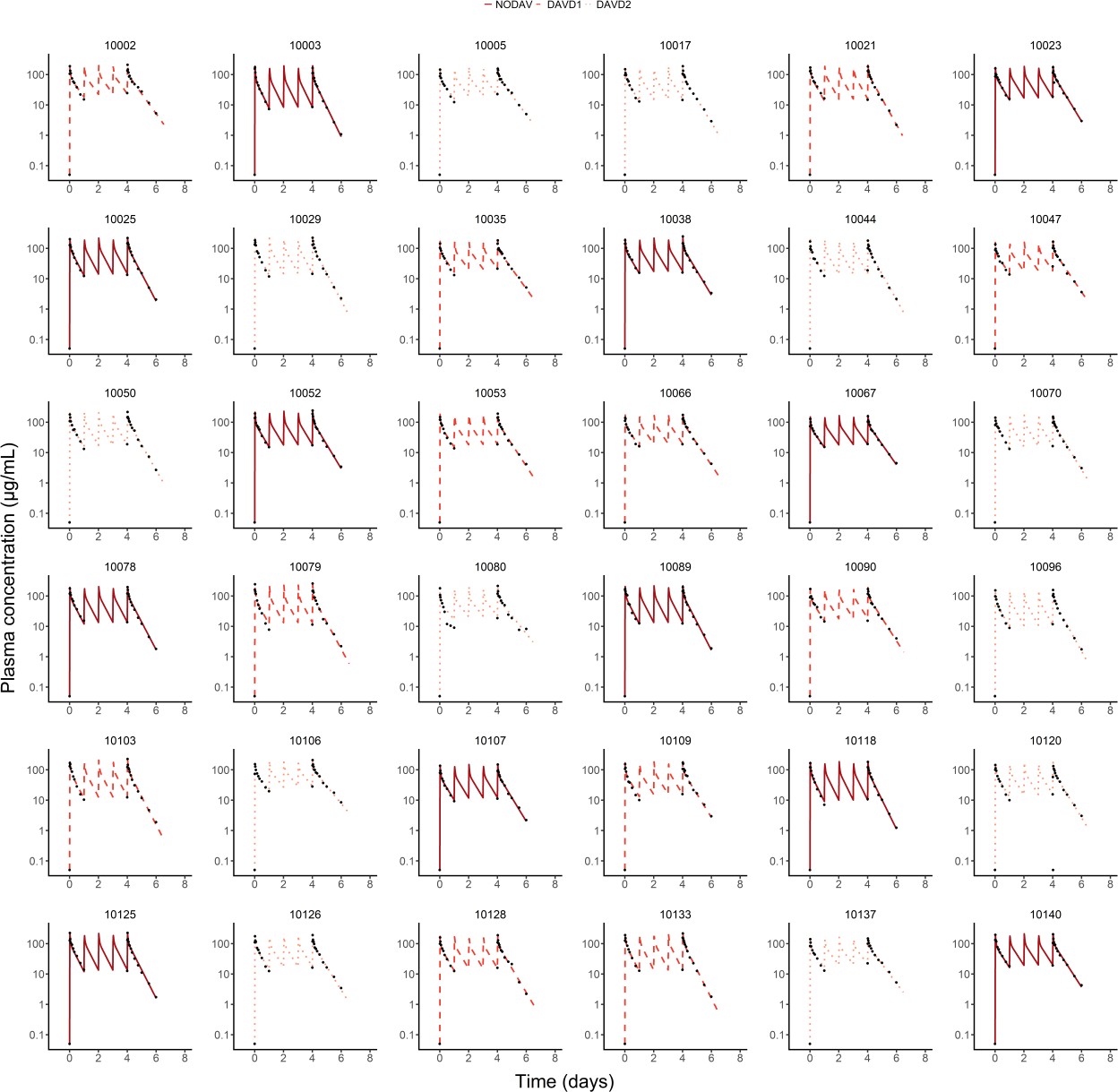


# Supplementary Figure S5

Individual fits for the fecal ceftriaxone concentrations for the final pharmacokinetic model in the 36 subjects treated with ceftriaxone. Black dots represent observed ceftriaxone concentrations. Red curves represent the individual pharmacokinetic profiles predicted by the model using estimated individual parameters. NODAV refers to subjects treated with ceftriaxone only (N=12); DAVD1 refers to subjects treated with ceftriaxone and the low dose of DAV132 (N=12); DAVD2 refers to subjects treated with ceftriaxone and the high dose of DAV132 (N=12).


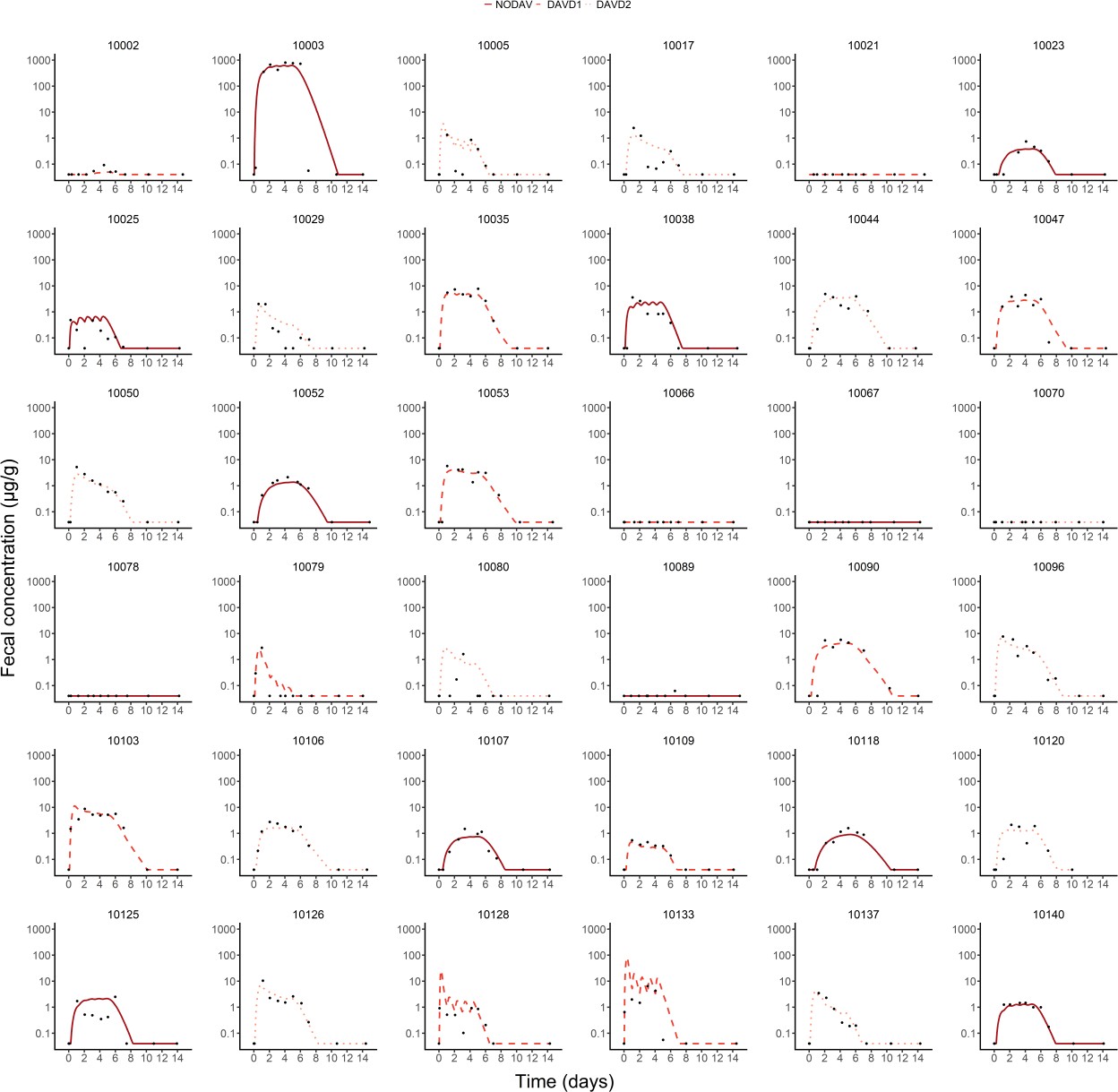


# Supplementary Figure S6

Individual fits for the plasma ceftazidime concentrations for the final pharmacokinetic model for the 36 subjects treated with ceftazidime/avibactam. Black dots represent observed ceftazidime concentrations. Blue curves represent the individual pharmacokinetic profiles predicted by the model using estimated individual parameters. NODAV refers to subjects treated with ceftazidime/avibactam only (N=12); DAVD1 refers to subjects treated with ceftazidime/avibactam and the low dose of DAV132 (N=12); DAVD2 refers to subjects treated with ceftazidime/avibactam and the high dose of DAV132 (N=12).


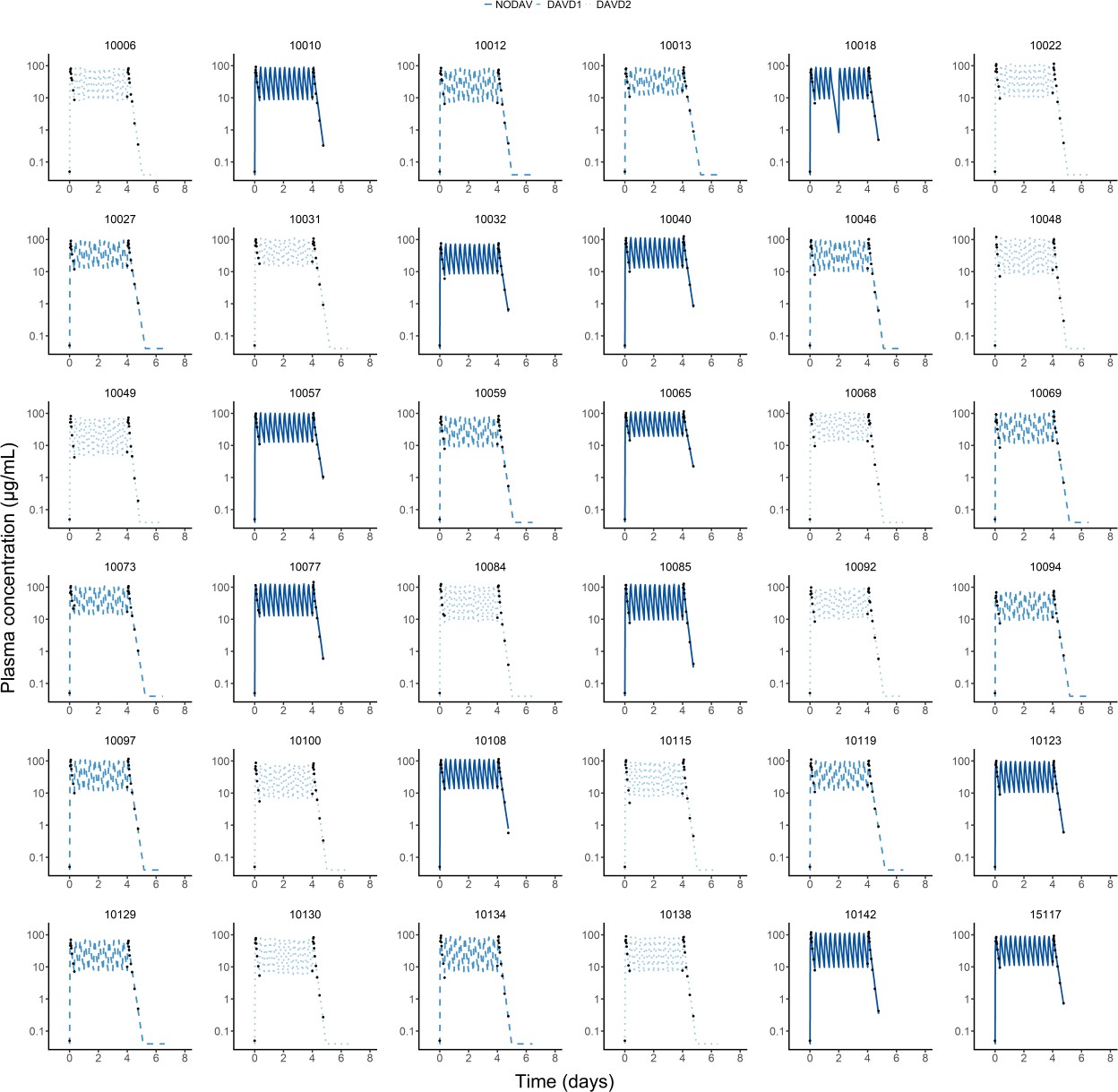


# Supplementary Figure S7

Individual fits for the fecal ceftazidime concentrations for the final pharmacokinetic model for the 36 subjects treated with ceftazidime/avibactam. Black dots represent observed ceftazidime concentrations. Blue curves represent the individual pharmacokinetic profiles predicted by the model using estimated individual parameters. NODAV refers to subjects treated with ceftazidime/avibactam only (N=12); DAVD1 refers to subjects treated with ceftazidime/avibactam and the low dose of DAV132 (N=12); DAVD2 refers to subjects treated with ceftazidime/avibactam and the high dose of DAV132 (N=12).


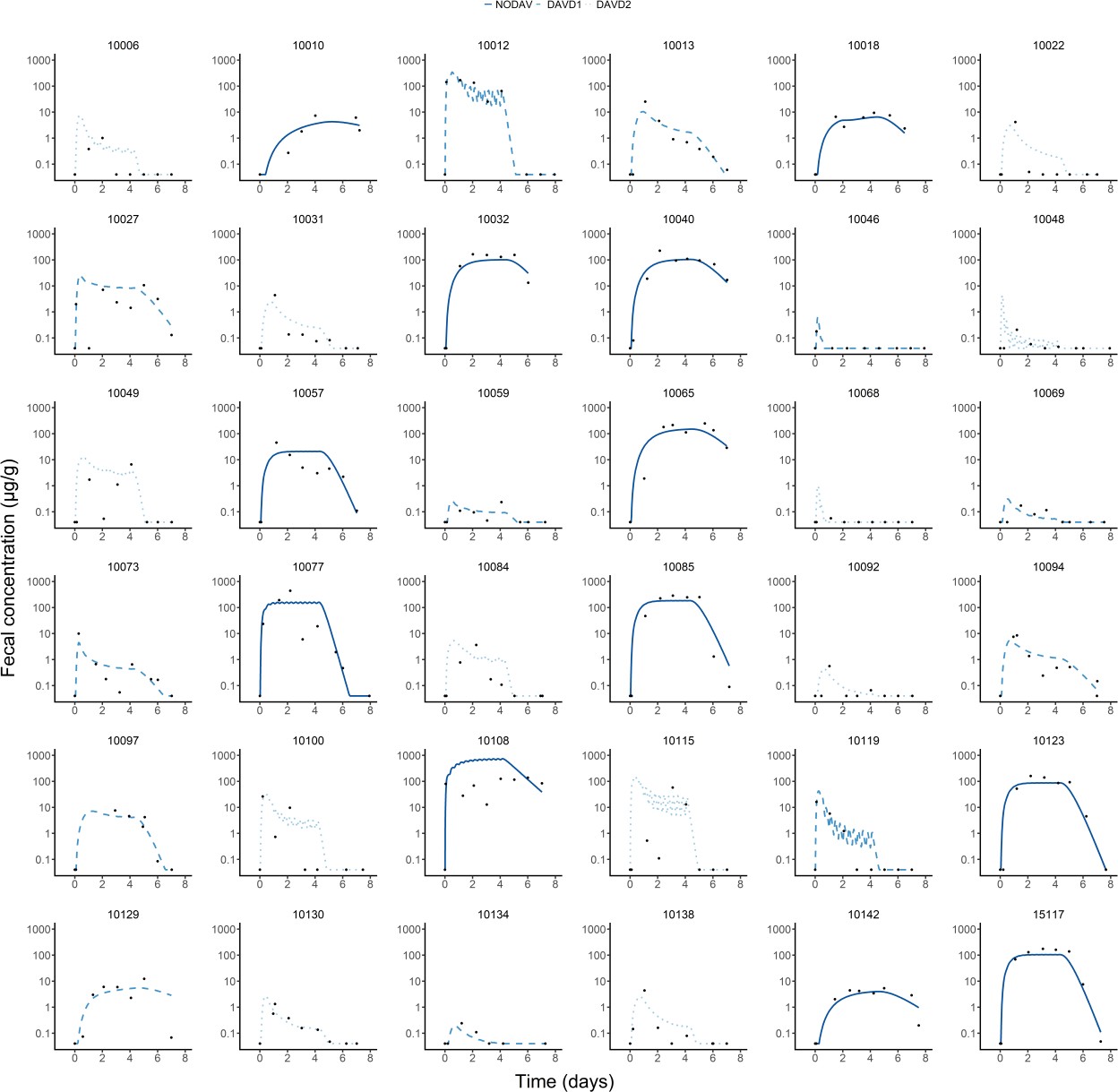


# Supplementary Figure S8

Individual fits for the plasma avibactam concentrations for the final pharmacokinetic model for the 36 subjects treated with ceftazidime/avibactam. Black dots represent observed avibactam concentrations. Purple curves represent the individual pharmacokinetic profiles predicted by the model using estimated individual parameters. NODAV refers to subjects treated with ceftazidime/avibactam only (N=12); DAVD1 refers to subjects treated with ceftazidime/avibactam and the low dose of DAV132 (N=12); DAVD2 refers to subjects treated with ceftazidime/avibactam and the high dose of DAV132 (N=12).


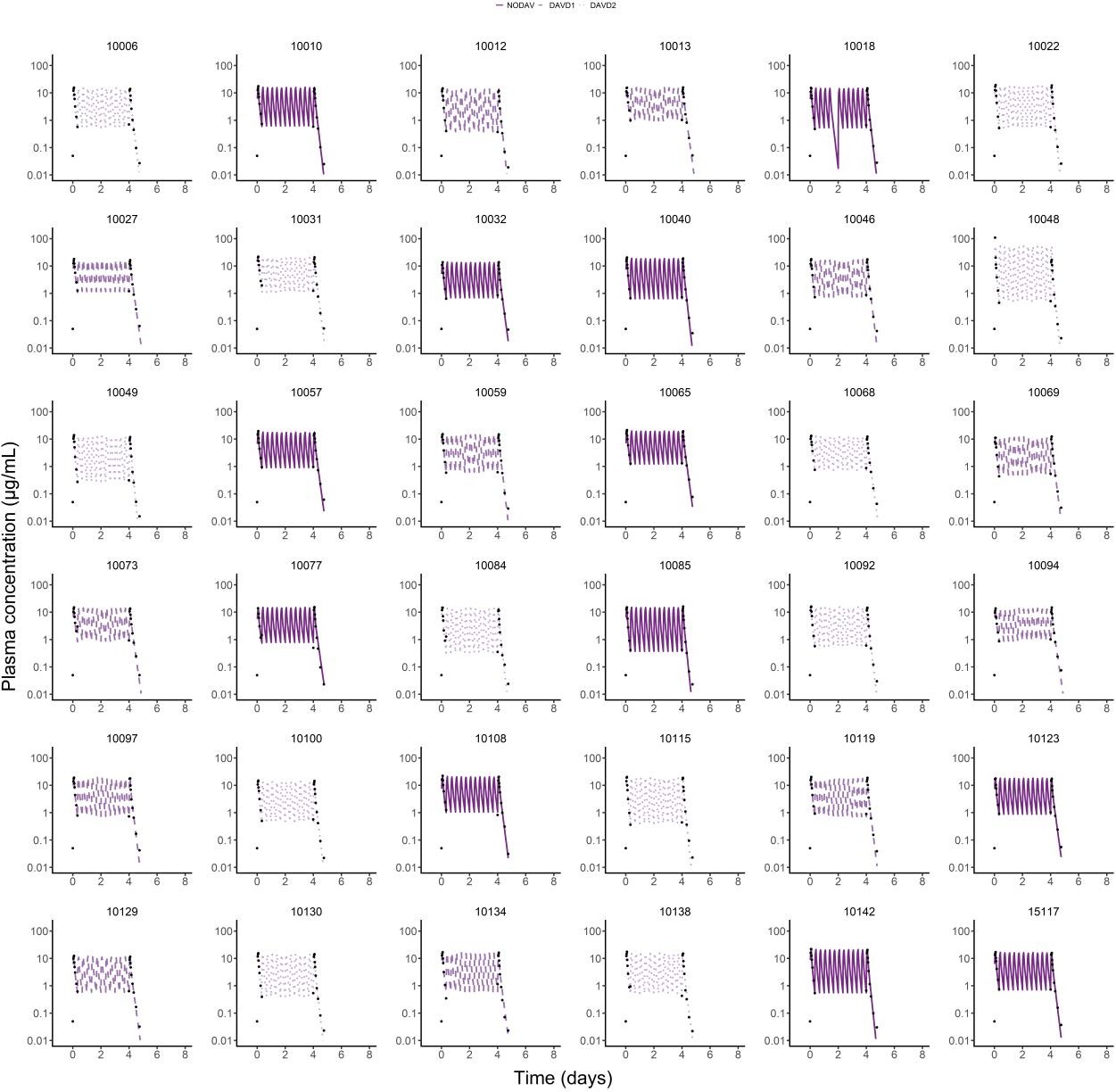


# Supplementary Figure S9

Individual fits for the fecal avibactam concentrations for the final pharmacokinetic model for the 36 subjects treated with ceftazidime/avibactam. Black dots represent observed avibactam concentrations. Purple curves represent the individual pharmacokinetic profiles predicted by the model using estimated individual parameters. NODAV refers to subjects treated with ceftazidime/avibactam only (N=12); DAVD1 refers to subjects treated with ceftazidime/avibactam and the low dose of DAV132 (N=12); DAVD2 refers to subjects treated with ceftazidime/avibactam and the high dose of DAV132 (N=12).


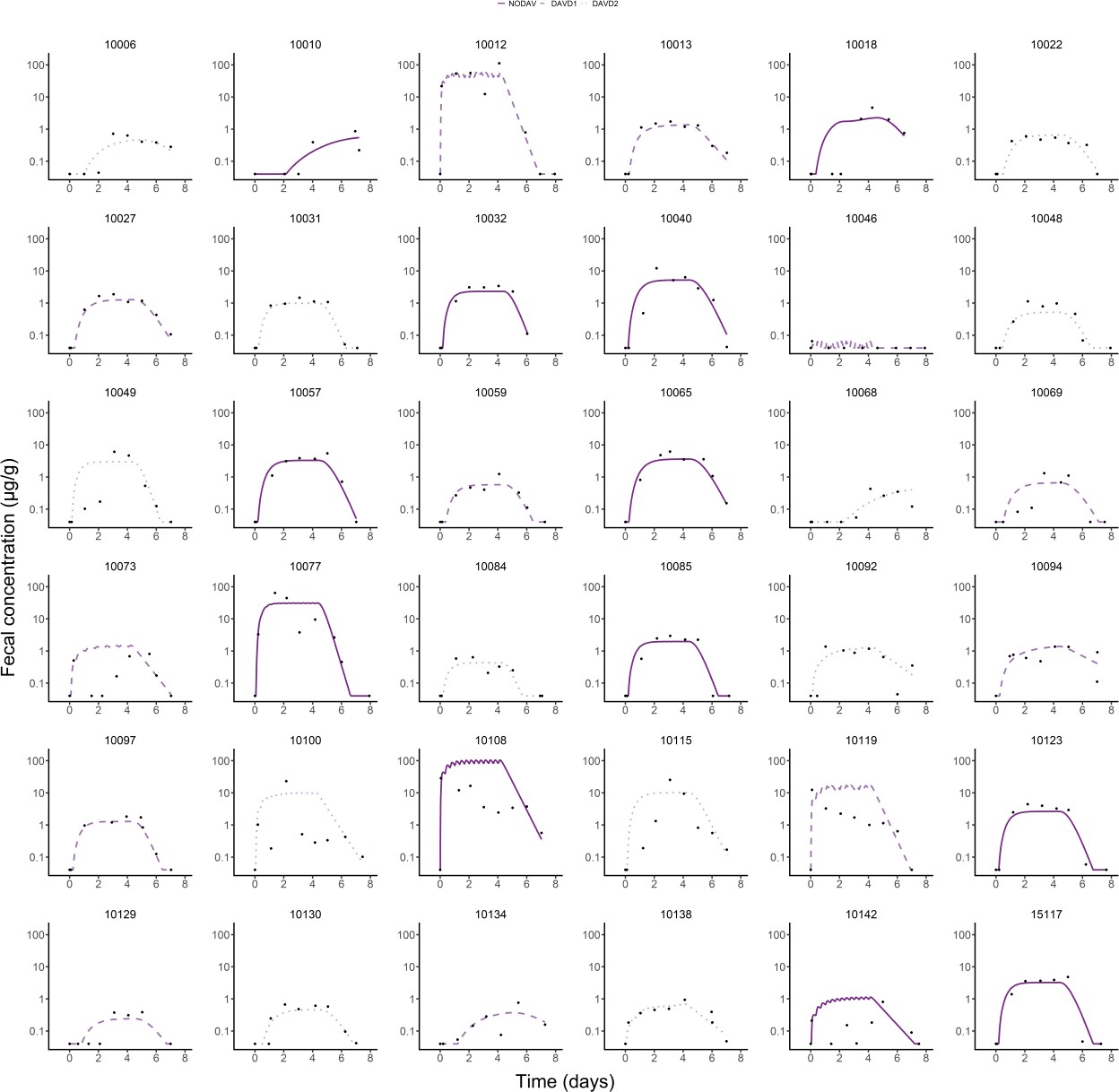


# Supplementary Figure S10

Individual fits for the plasma piperacillin concentrations for the final pharmacokinetic model for the 37 subjects treated with piperacillin/tazobactam. Black dots represent observed piperacillin concentrations. Green curves represent the individual pharmacokinetic profiles predicted by the model using estimated individual parameters. NODAV refers to subjects treated with piperacillin/tazobactam only (N=12); DAVD1 refers to subjects treated with piperacillin/tazobactam and the low dose of DAV132 (N=12); DAVD2 refers to subjects treated with piperacillin/tazobactam and the high dose of DAV132 (N=13).


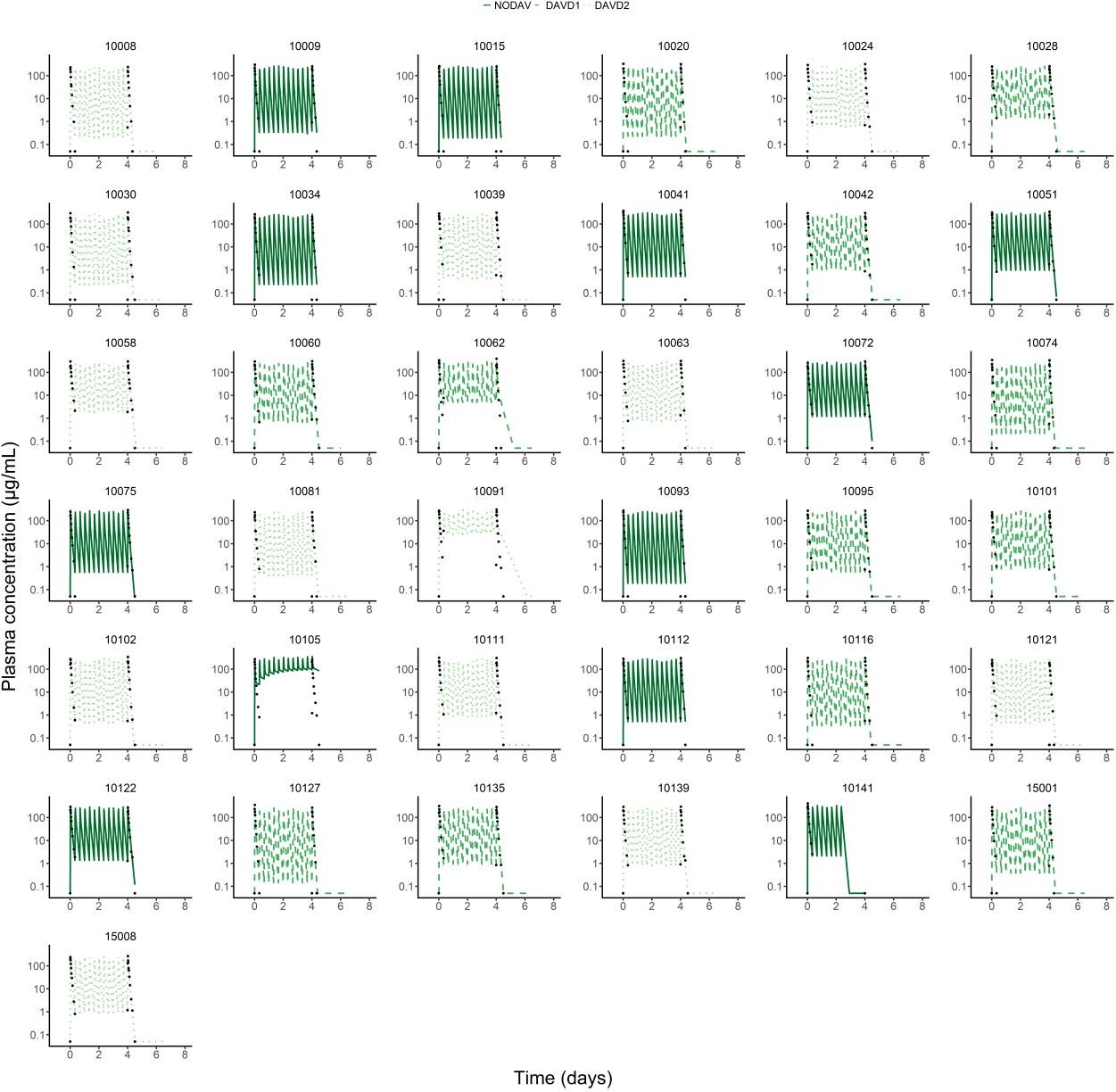


# Supplementary Figure S11

Individual fits for the fecal piperacillin concentrations for the final pharmacokinetic model for the 37 subjects treated with piperacillin/tazobactam. Black dots represent observed piperacillin concentrations. Green curves represent the individual pharmacokinetic profiles predicted by the model using estimated individual parameters. NODAV refers to subjects treated with piperacillin/tazobactam only (N=12); DAVD1 refers to subjects treated with piperacillin/tazobactam and the low dose of DAV132 (N=12); DAVD2 refers to subjects treated with piperacillin/tazobactam and the high dose of DAV132 (N=13).


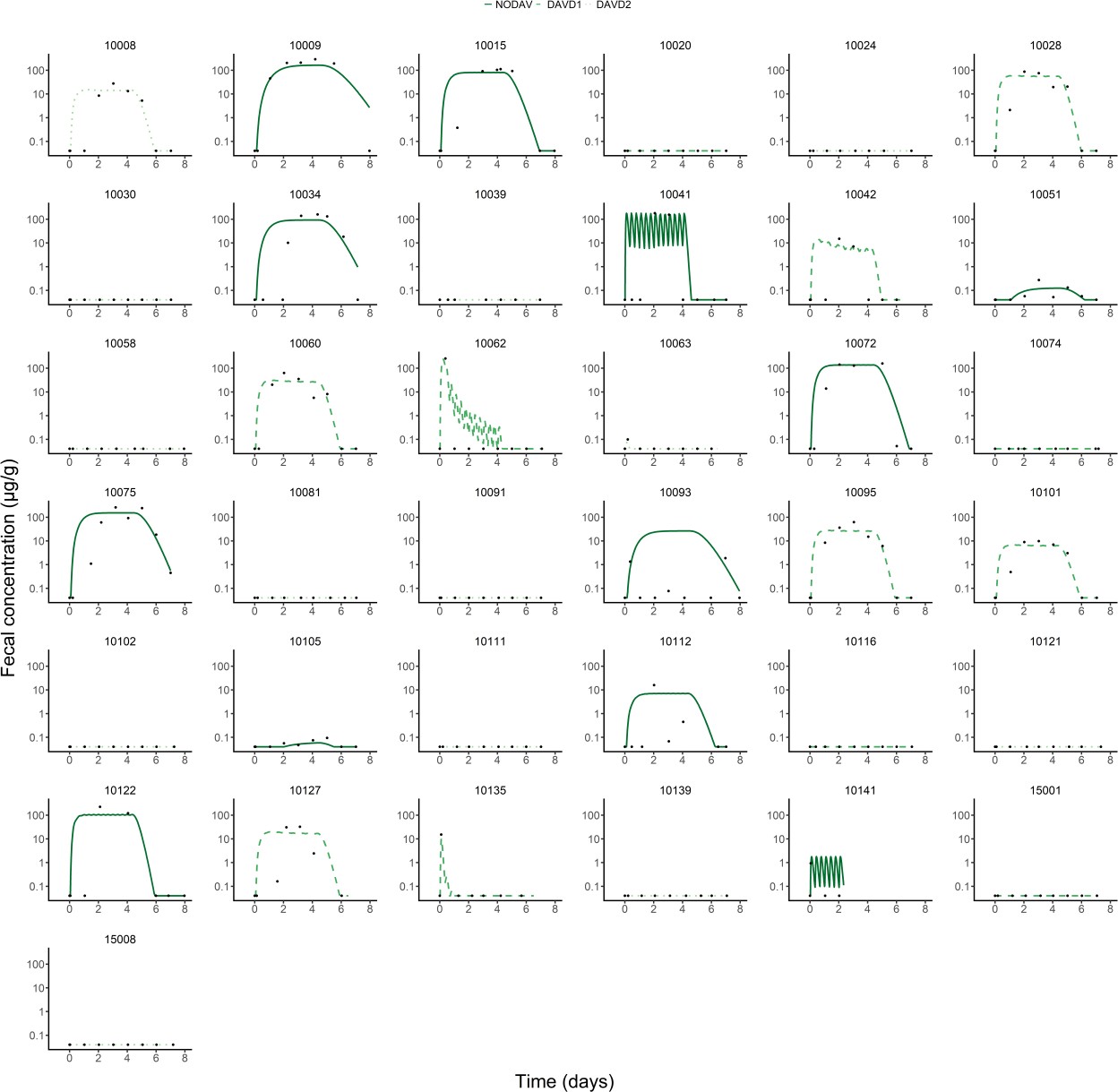


# Supplementary Figure S12

Individual fits for the plasma tazobactam concentrations for the final pharmacokinetic model for the 37 subjects treated with piperacillin/tazobactam. Black dots represent observed tazobactam concentrations. Brown curves represent the individual pharmacokinetic profiles predicted by the model using estimated individual parameters. NODAV refers to subjects treated with piperacillin/tazobactam only (N=12); DAVD1 refers to subjects treated with piperacillin/tazobactam and the low dose of DAV132 (N=12); DAVD2 refers to subjects treated with piperacillin/tazobactam and the high dose of DAV132 (N=13).


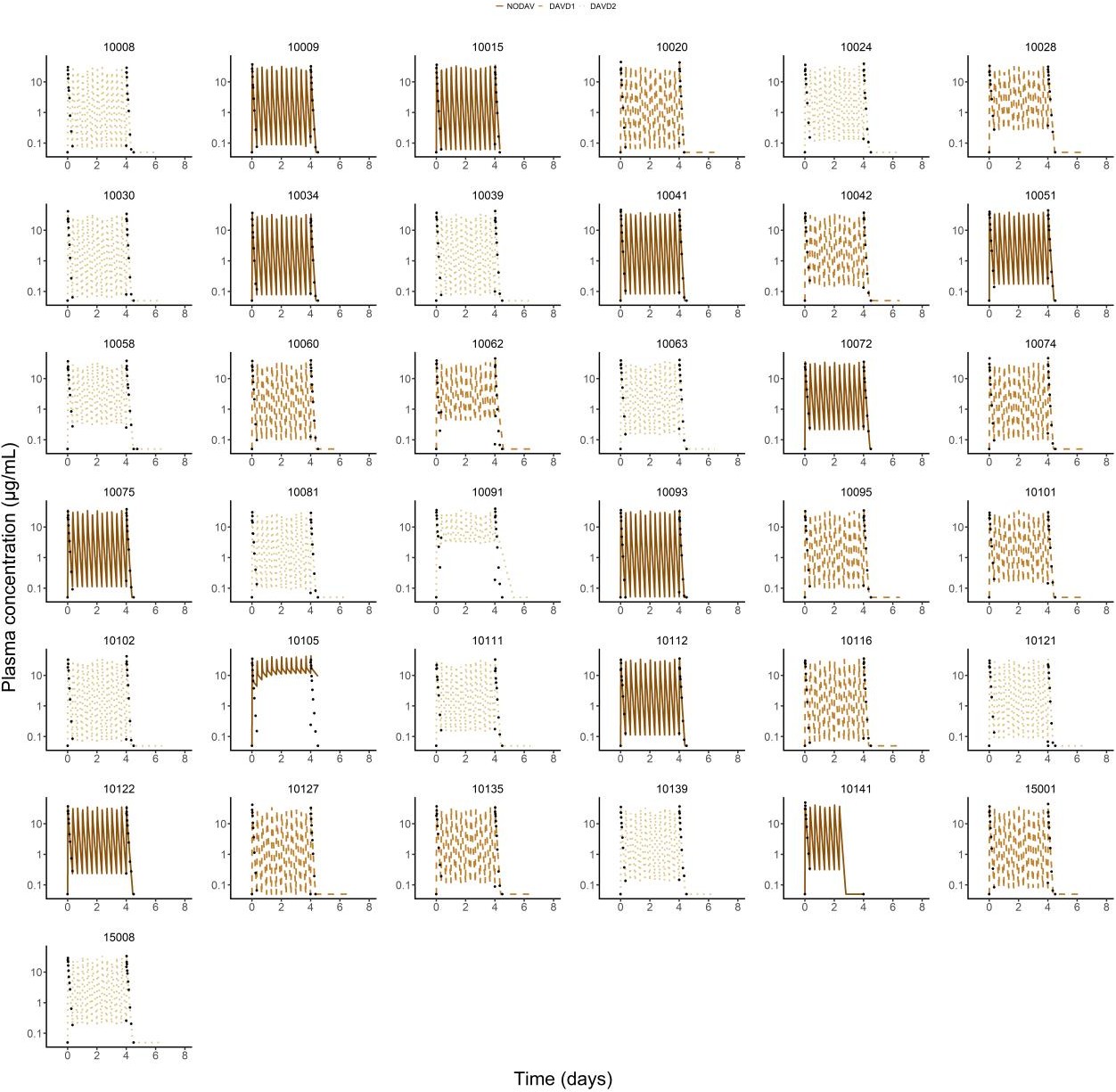


# Supplementary Figure S13

Individual fits for the fecal tazobactam concentrations for the final pharmacokinetic model for the 37 subjects treated with piperacillin/tazobactam. Black dots represent observed tazobactam concentrations. Brown curves represent the individual pharmacokinetic profiles predicted by the model using estimated individual parameters. NODAV refers to subjects treated with piperacillin/tazobactam only (N=12); DAVD1 refers to subjects treated with piperacillin/tazobactam and the low dose of DAV132 (N=12); DAVD2 refers to subjects treated with piperacillin/tazobactam and the high dose of DAV132 (N=13).


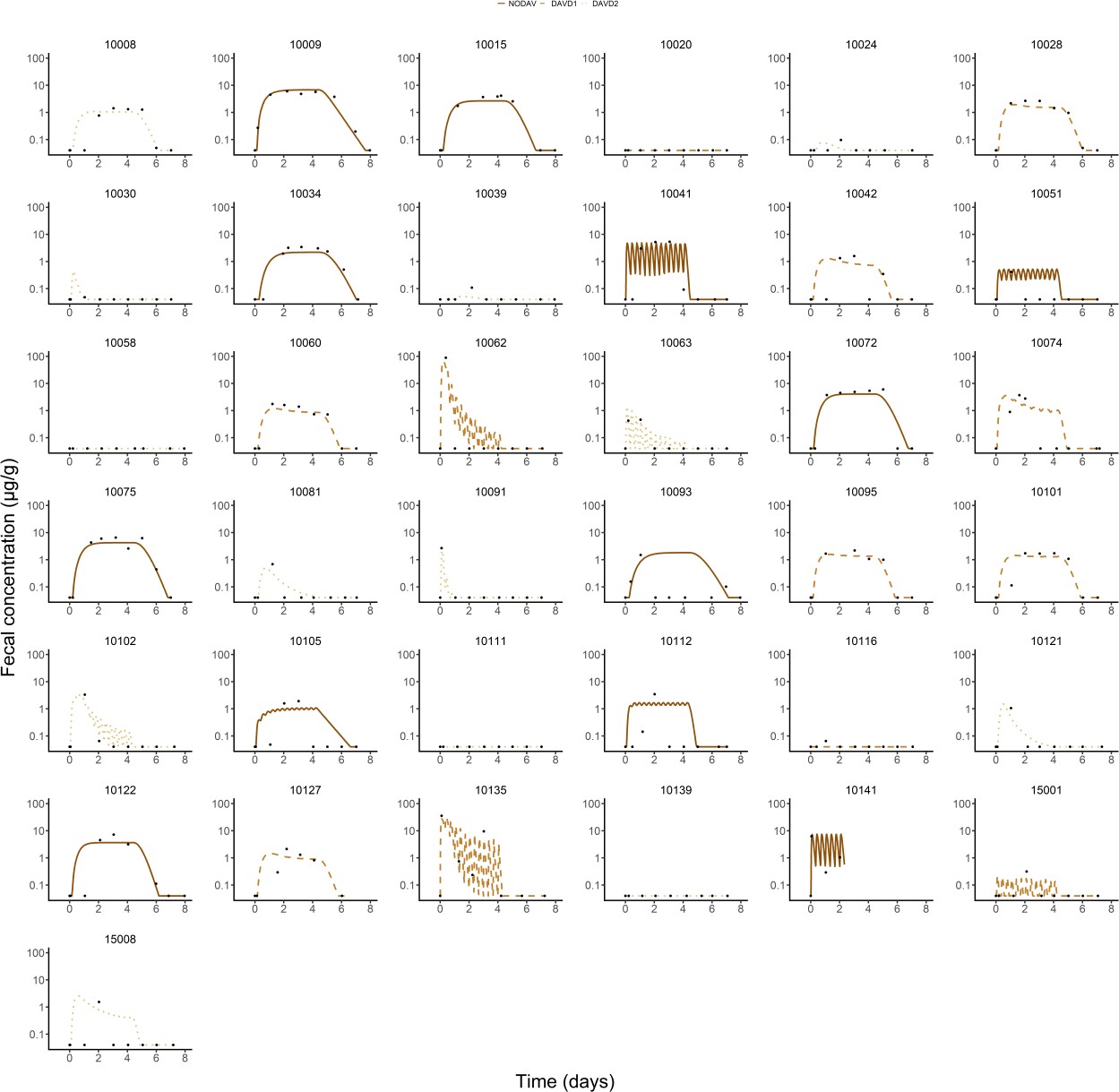


# Supplementary Figure S14

Normalized predicted distribution errors (NPDE) versus time and NPDE versus prediction for the final plasma pharmacokinetic models.


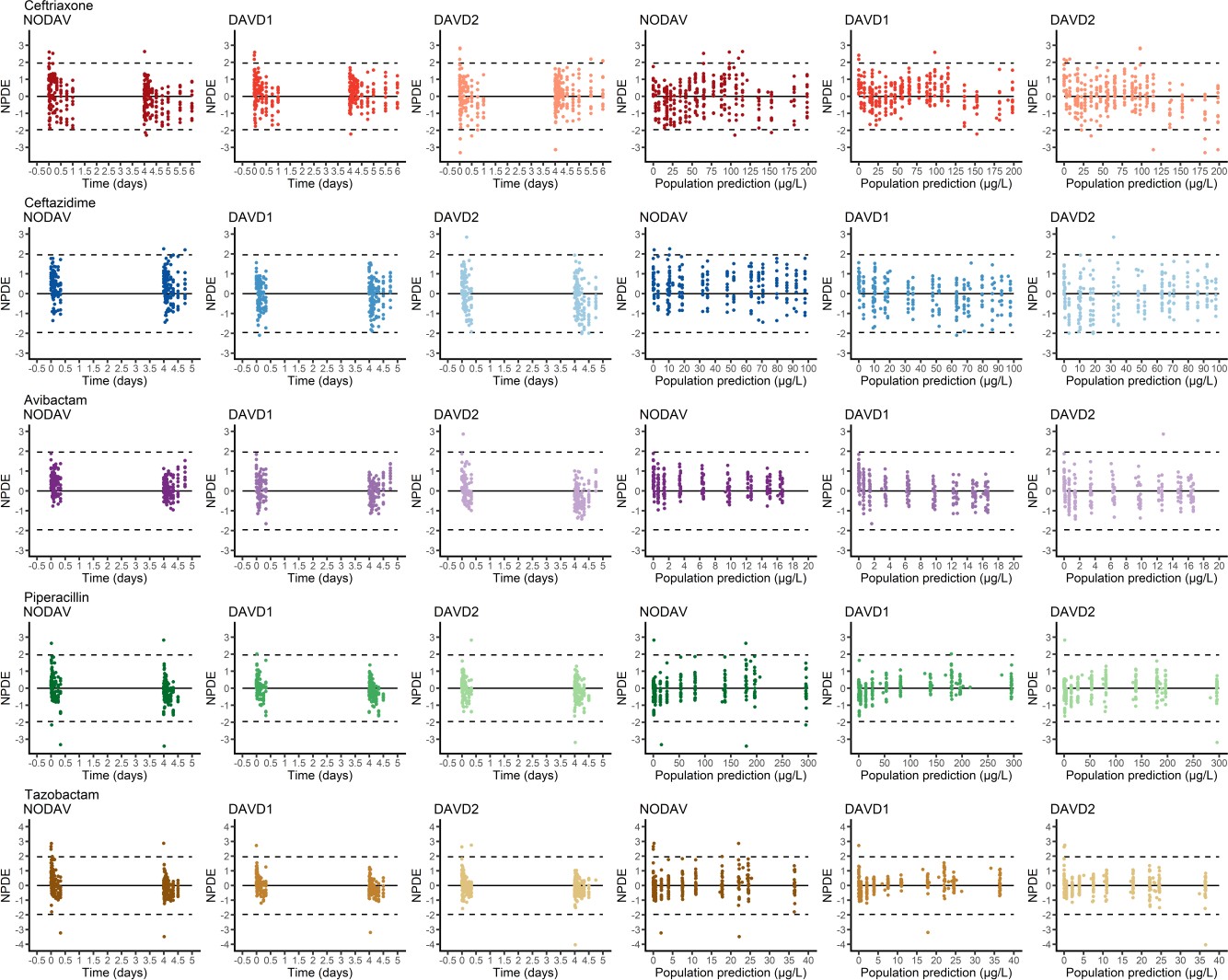
NODAV refers to subjects treated with antibiotic only; DAVD1 refers to subjects treated with antibiotic and the low dose of DAV132; DAVD2 refers to subjects treated with antibiotic and the high dose of DAV132.

# Supplementary Figure S15

Normalized predicted distribution errors (NPDE) versus time and NPDE versus prediction for the final fecal pharmacokinetic models.


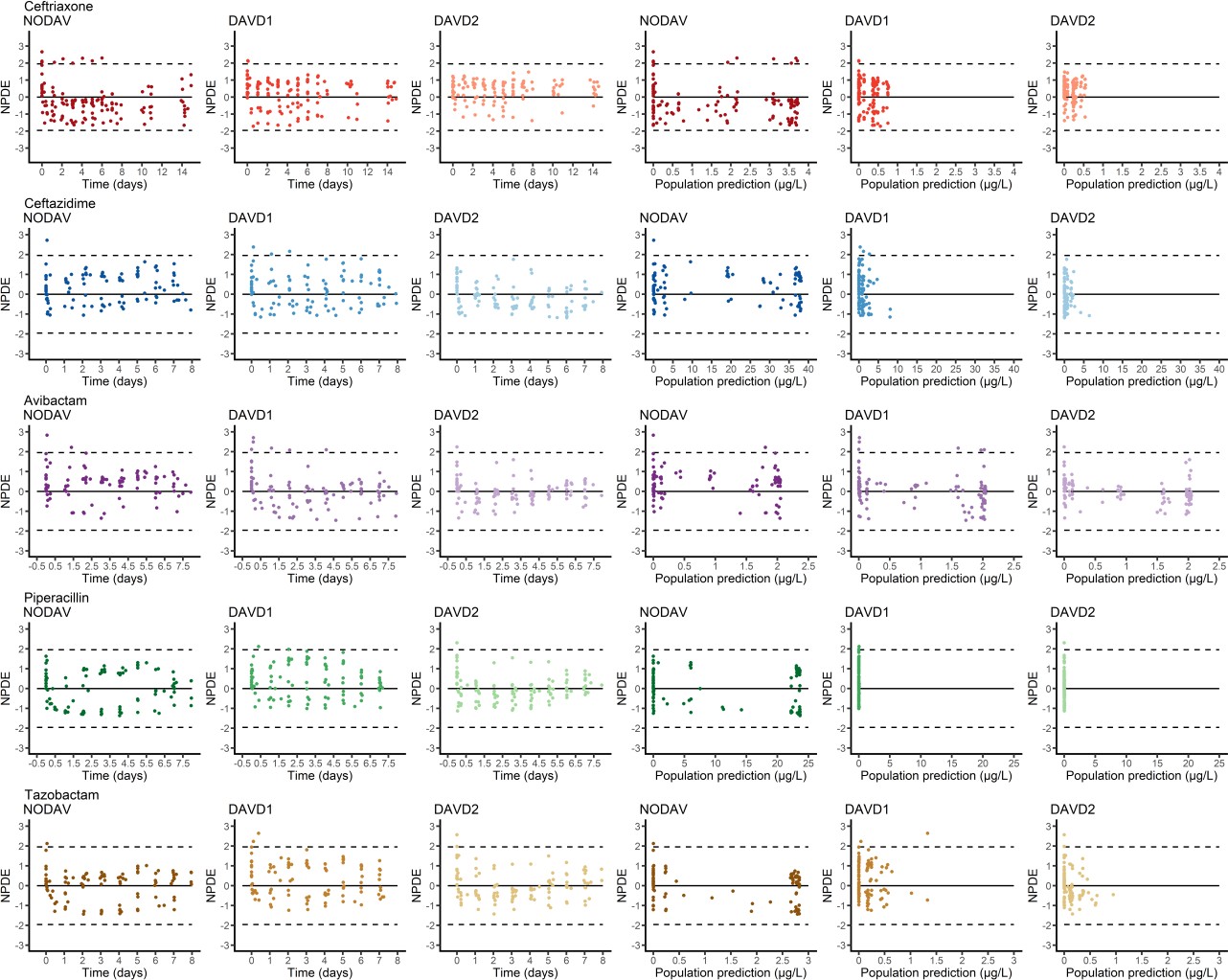
NODAV refers to subjects treated with antibiotic only; DAVD1 refers to subjects treated with antibiotic and the low dose of DAV132; DAVD2 refers to subjects treated with antibiotic and the high dose of DAV132.

# Supplementary Figure S16

Spaghetti plot of the gut bacterial diversity in the 142 subjects included in the analysis in the control (panel A), ceftriaxone (panel B), ceftazidime/avibactam (panel C) and piperacillin/tazobactam (panel D) groups. NODAV refers to subjects treated with antibiotic only; DAVD1 refers to subjects treated with antibiotic and the low dose of DAV132; DAVD2 refers to subjects treated with antibiotic and the high dose of DAV132.


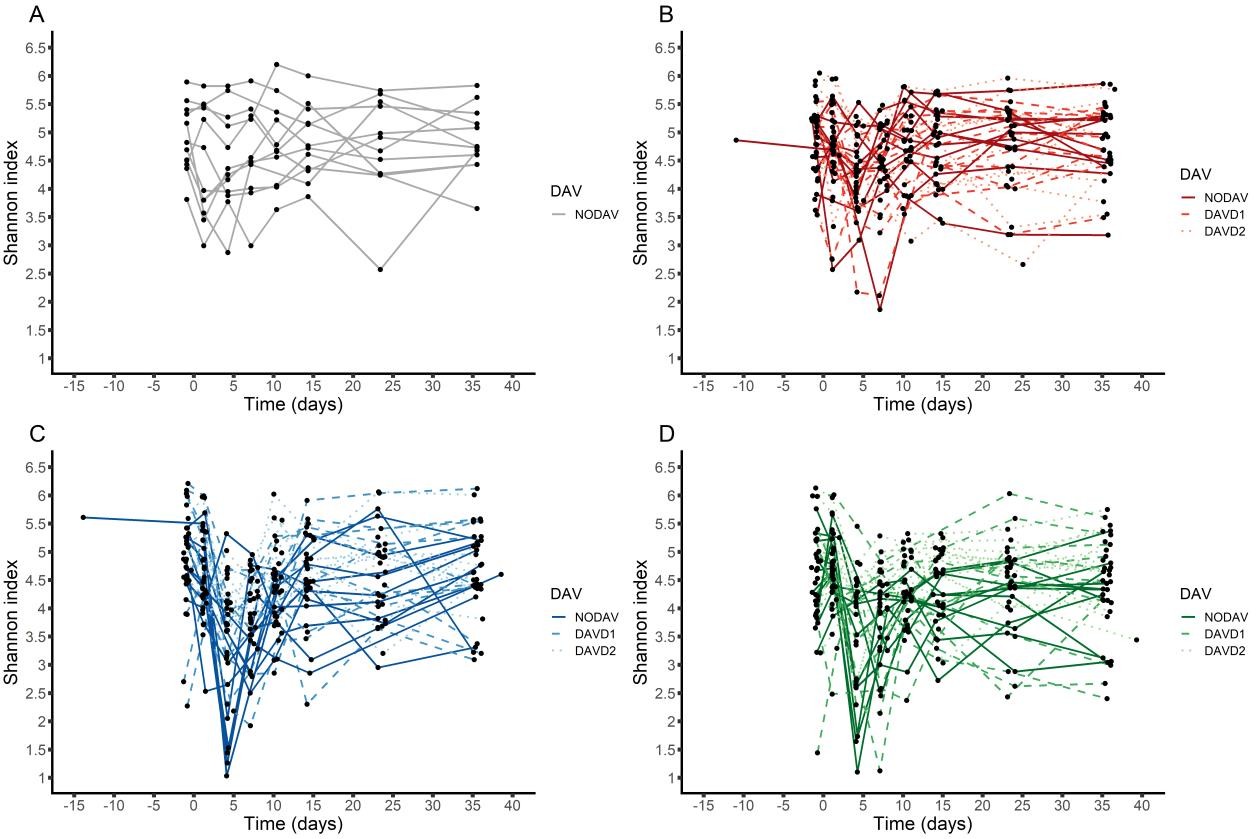


# Supplementary Figure S17

Individual fits for the final pharmacodynamic model for the 121 subjects included in the analysis. Black dots represent observed Shannon index values. Red curves depict ceftriaxone-treated subjects. Blue curves depict ceftazidime/avibactam-treated subjects. Green curves depict piperacillin/tazobactam-treated subjects. Grey curves depict untreated control subjects. NODAV refers to subjects treated with antibiotic only; DAVD1 refers to subjects treated with antibiotic and the low dose of DAV132; DAVD2 refers to subjects treated with antibiotic and the high dose of DAV132.


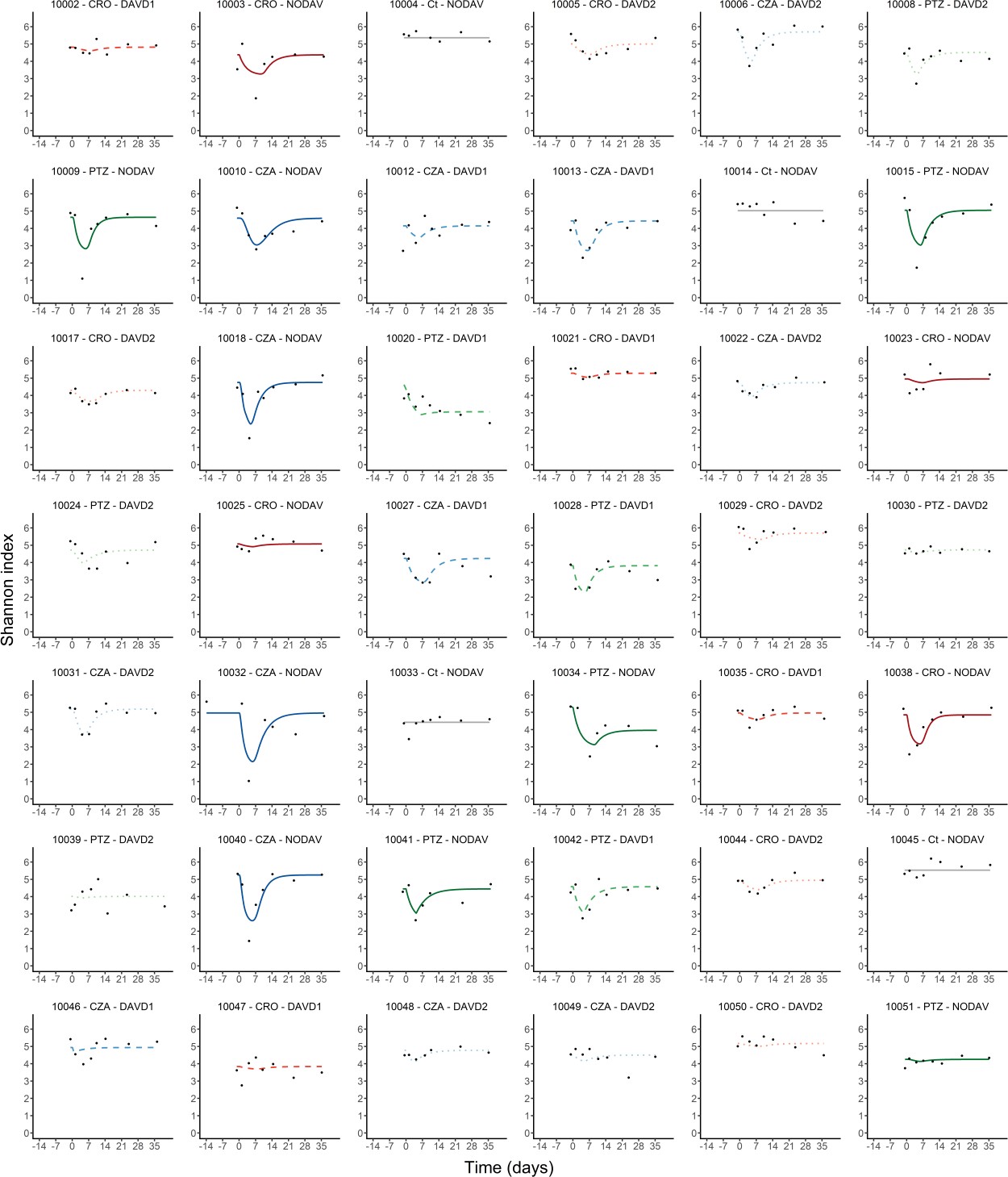


# Supplementary Figure S17 (continued)

Individual fits for the final pharmacodynamic model for the 121 subjects included in the analysis. Black dots represent observed Shannon index values. Red curves depict ceftriaxone-treated subjects. Blue curves depict ceftazidime/avibactam-treated subjects. Green curves depict piperacillin/tazobactam-treated subjects. Grey curves depict untreated control subjects. NODAV refers to subjects treated with antibiotic only; DAVD1 refers to subjects treated with antibiotic and the low dose of DAV132; DAVD2 refers to subjects treated with antibiotic and the high dose of DAV132.


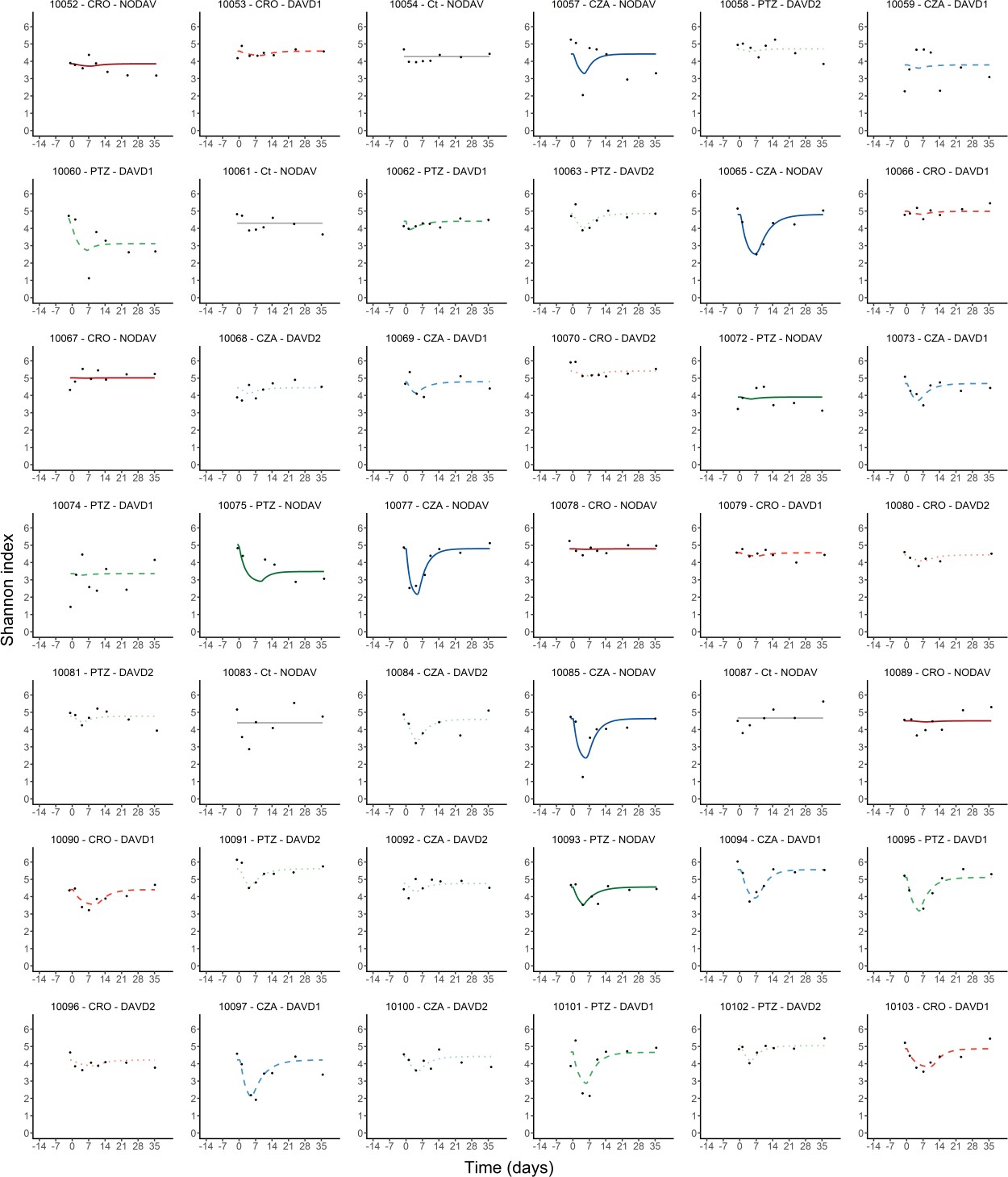


# Supplementary Figure S17 (continued)


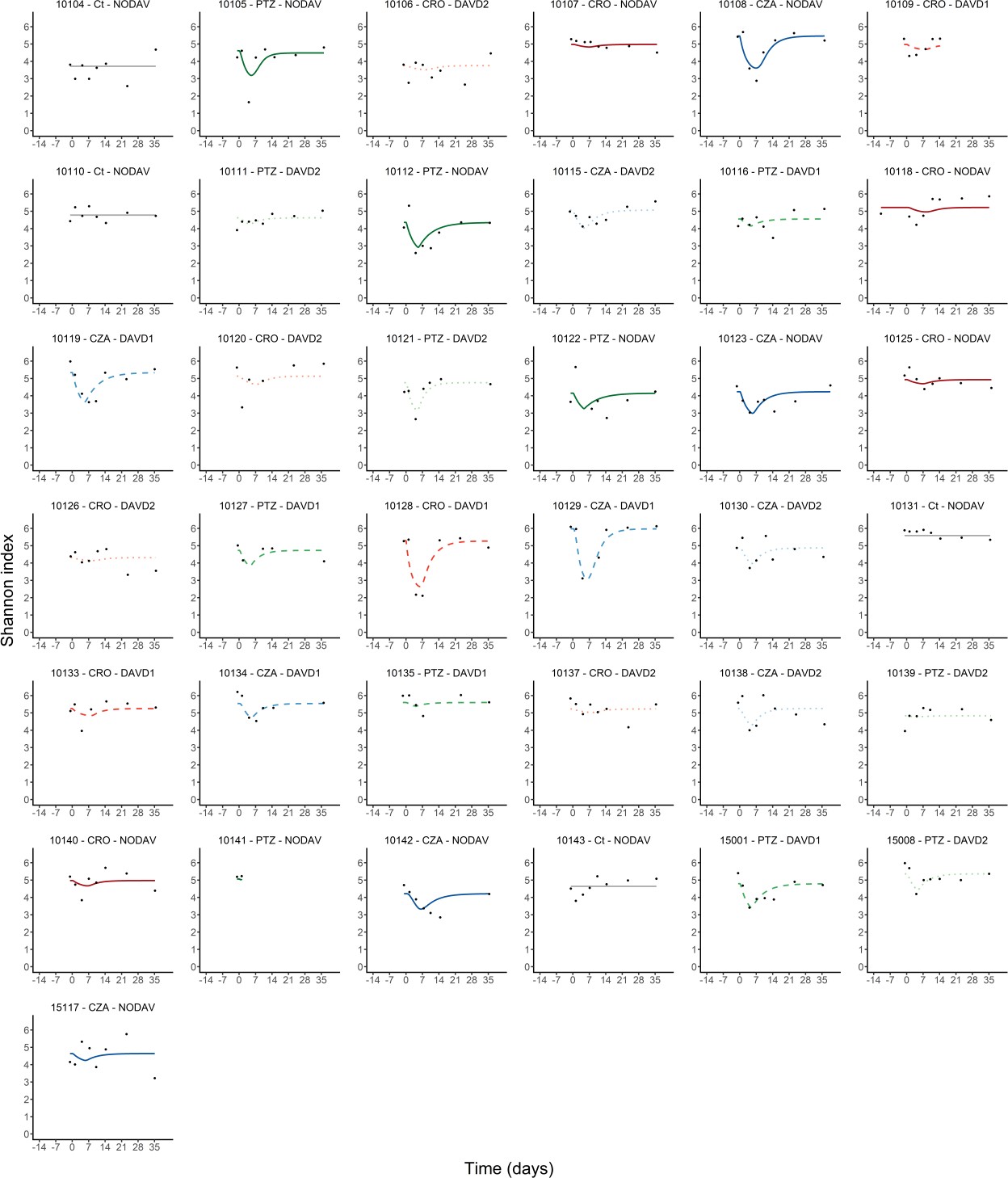
Individual fits for the final pharmacodynamic model for the 121 subjects included in the analysis. Black dots represent observed Shannon index values. Red curves depict ceftriaxone-treated subjects. Blue curves depict ceftazidime/avibactam-treated subjects. Green curves depict piperacillin/tazobactam-treated subjects. Grey curves depict untreated control subjects. NODAV refers to subjects treated with antibiotic only; DAVD1 refers to subjects treated with antibiotic and the low dose of DAV132; DAVD2 refers to subjects treated with antibiotic and the high dose of DAV132.

# Supplementary Figure S18

Normalized predicted distribution errors (NPDE) versus time and NPDE versus prediction for the final fecal pharmacodynamic model.


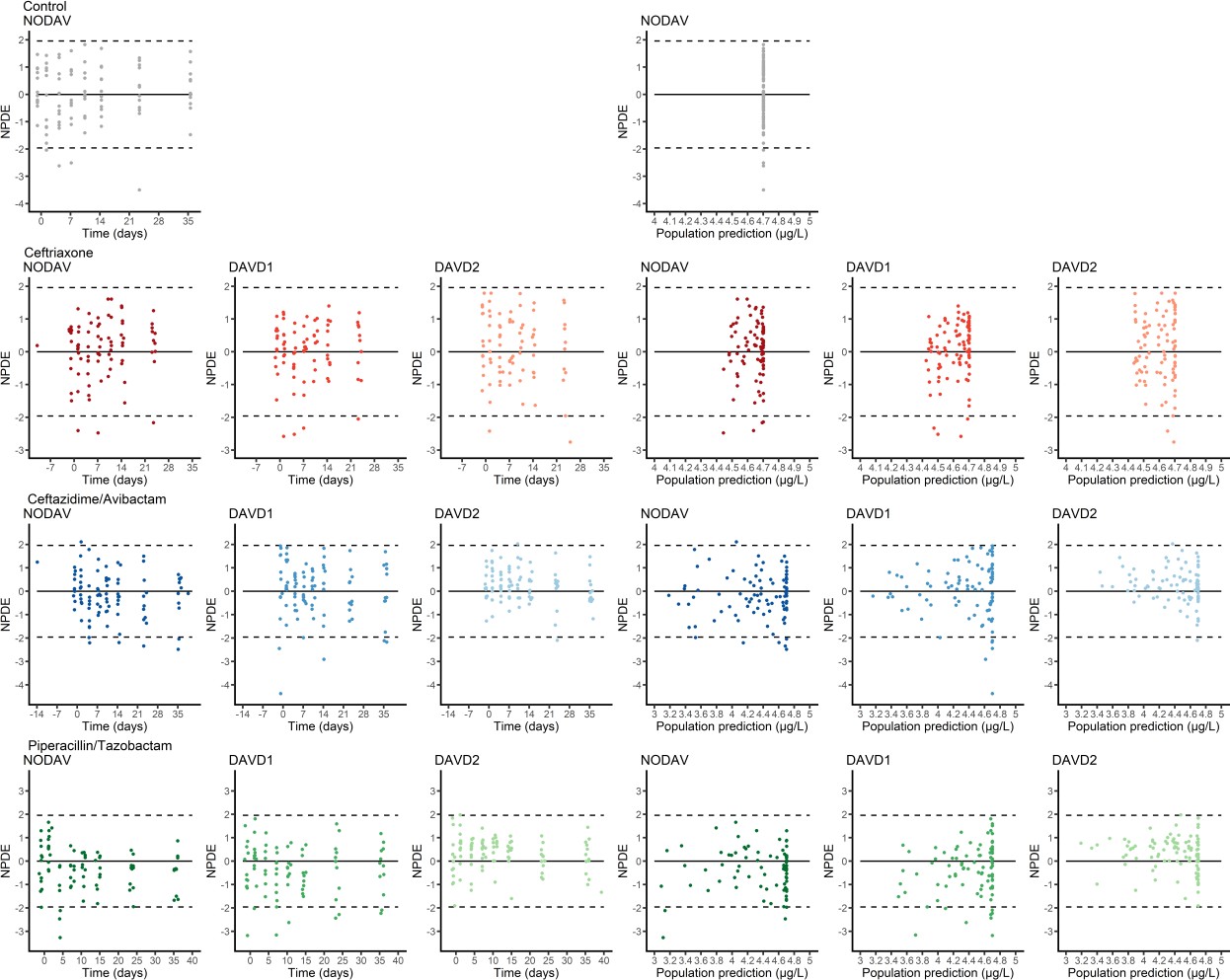
NODAV refers to subjects treated with antibiotic only; DAVD1 refers to subjects treated with antibiotic and the low dose of DAV132; DAVD2 refers to subjects treated with antibiotic and the high dose of DAV132.

# Supplementary Figure S19


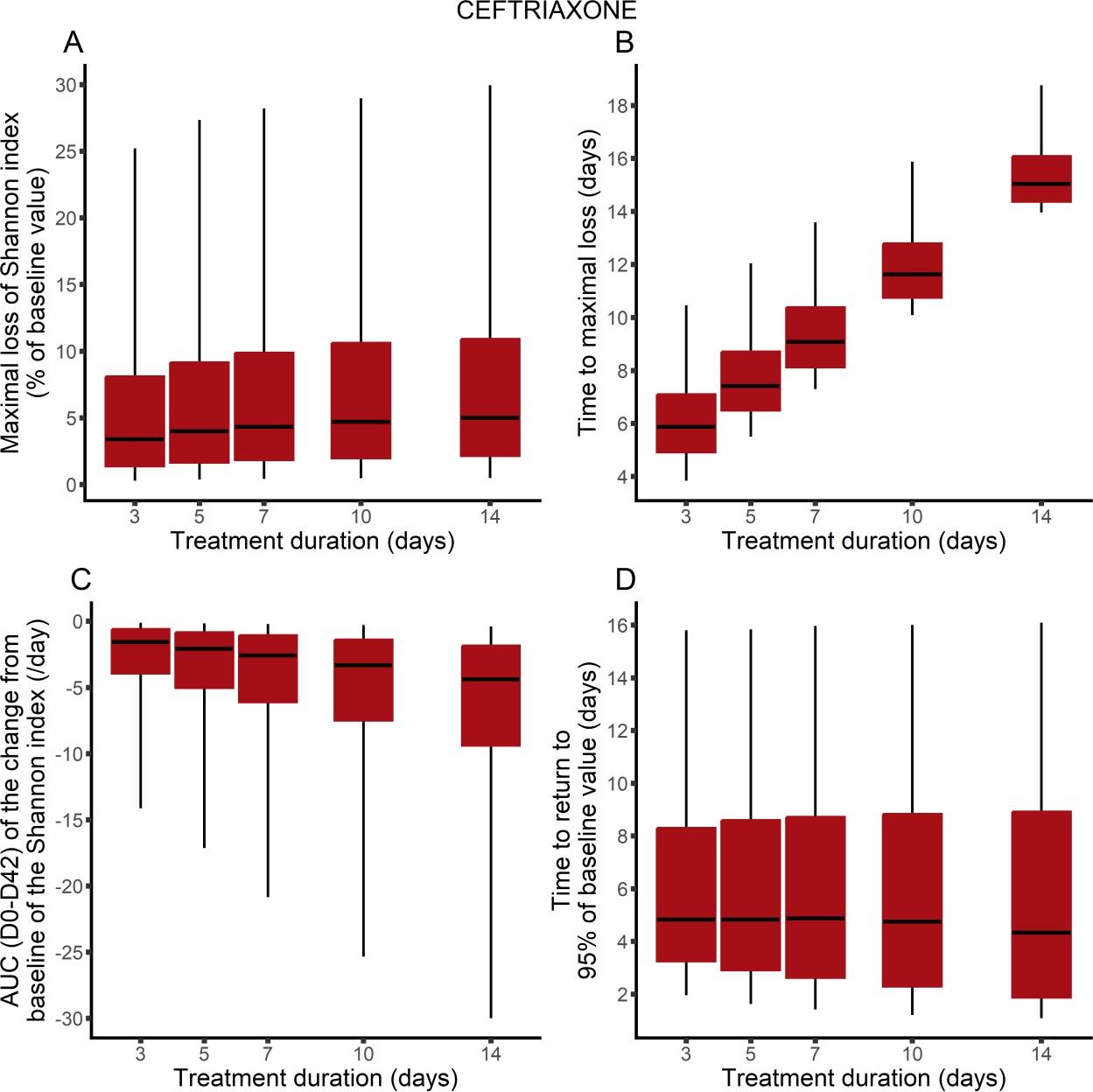
Derived indices of the impact of ceftriaxone on bacterial gut microbiota following various treatment durations, obtained from 1000 individuals simulated in the asymptotic distribution of the parameters. The boxes present the 25th and 75th percentiles and the horizontal black bar reports the median value, while whiskers report 10th and 90th percentiles.

# Supplementary Figure S20


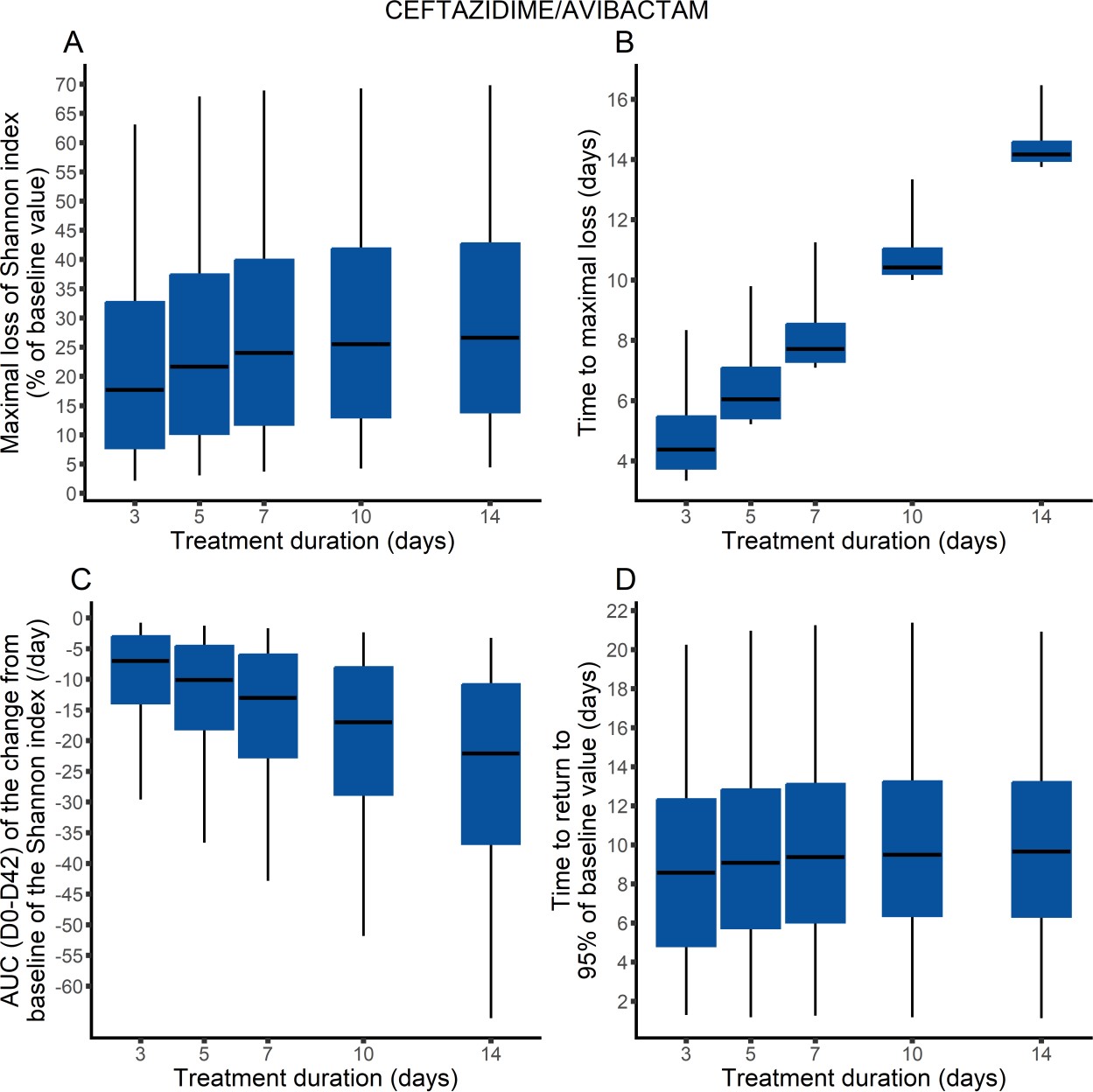
Derived indices of the impact of ceftazidime/avibactam on bacterial gut microbiota following various treatment durations, obtained from 1000 individuals simulated in the asymptotic distribution of the parameters. The boxes present the 25th and 75th percentiles and the horizontal black bar reports the median value, while whiskers report 10th and 90th percentiles.

# Supplementary Figure S21


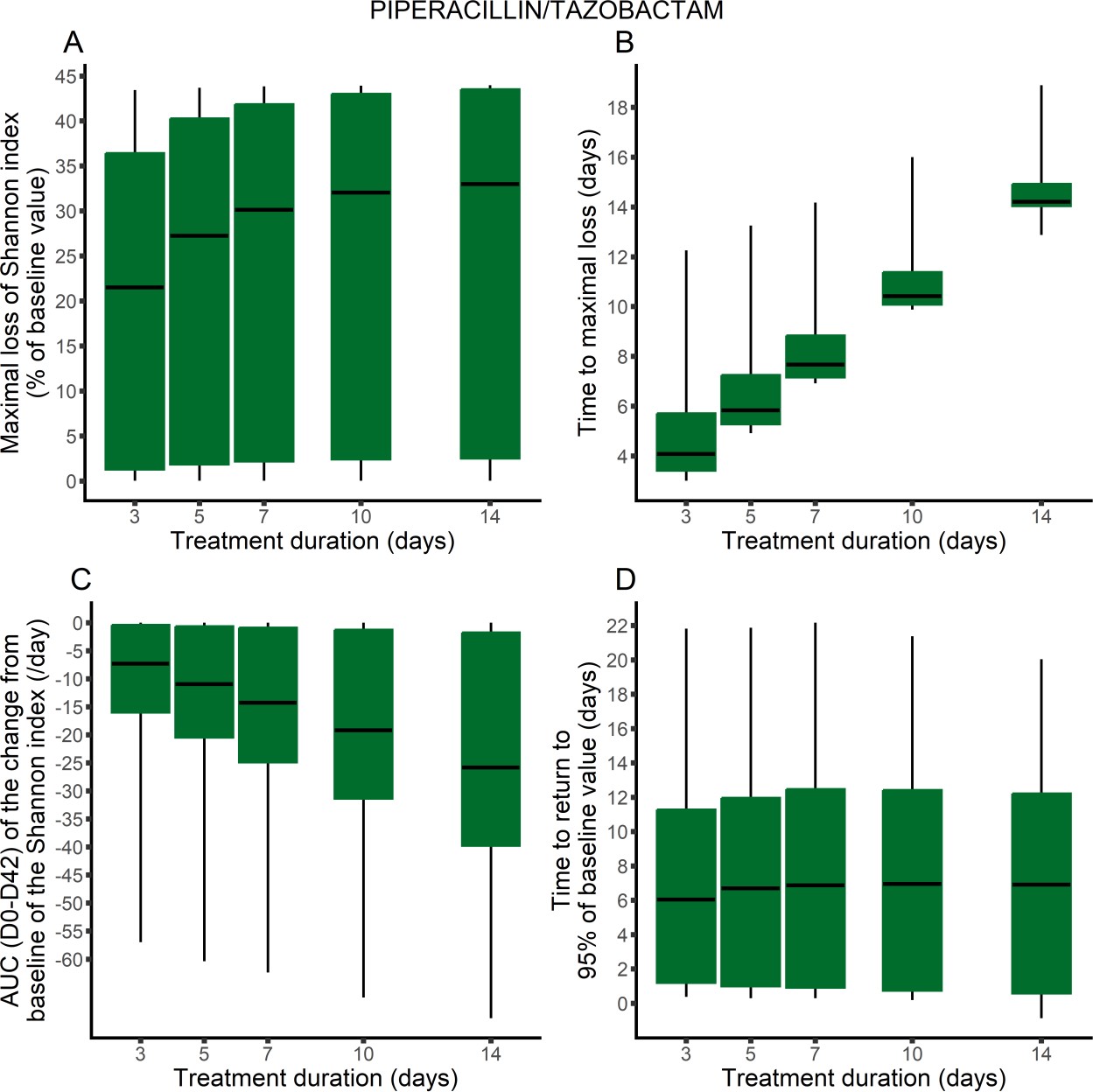
Derived indices of the impact of piperacillin/tazobactam on bacterial gut microbiota following various treatment durations, obtained from 1000 individuals simulated in the asymptotic distribution of the parameters. The boxes present the 25th and 75th percentiles and the horizontal black bar reports the median value, while whiskers report 10th and 90th percentiles.

# Supplementary Figure S22


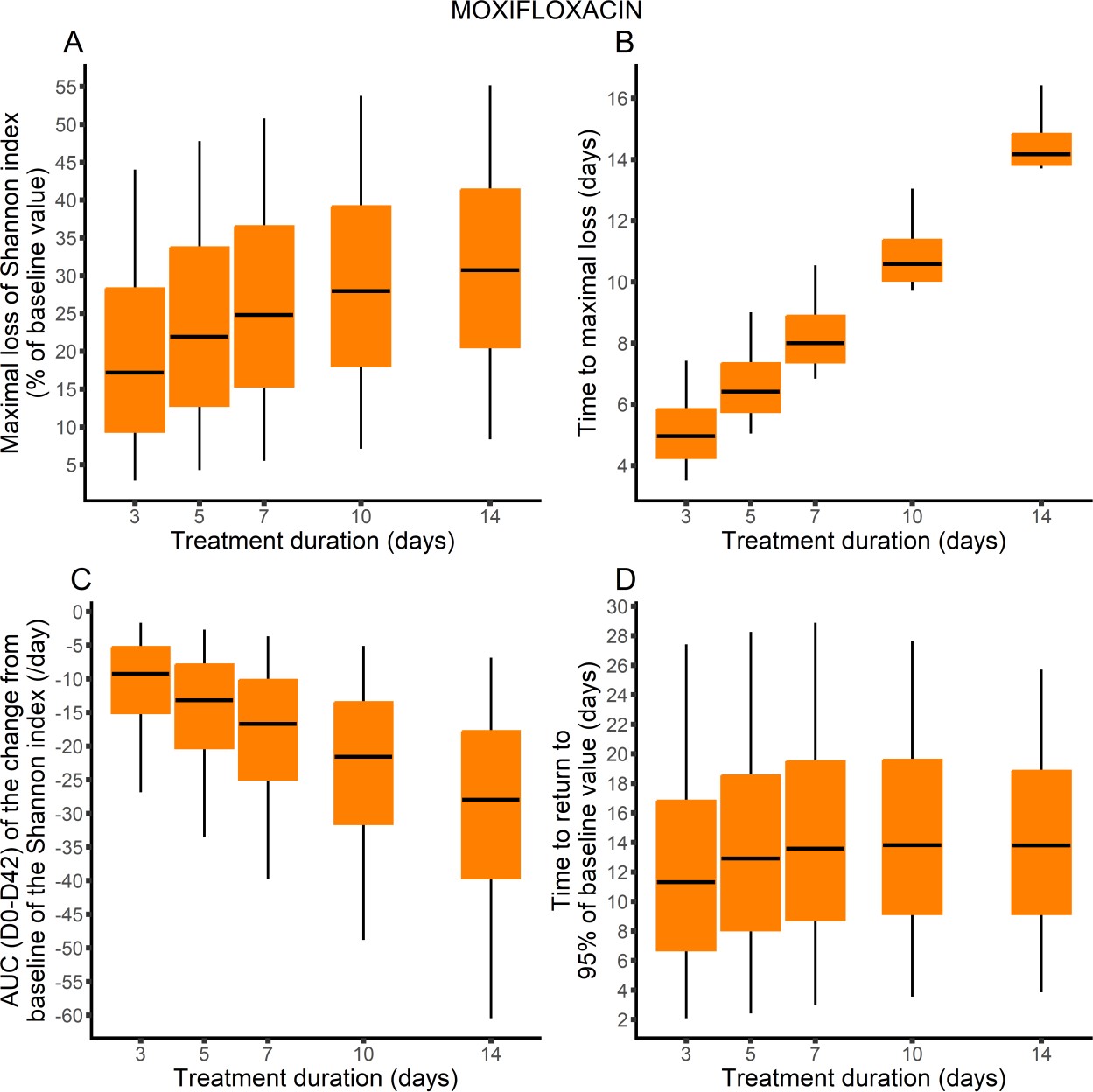
Derived indices of the impact of moxifloxacin on bacterial gut microbiota following various treatment durations, obtained from 1000 individuals simulated in the asymptotic distribution of the parameters. The boxes present the 25th and 75th percentiles and the horizontal black bar reports the median value, while whiskers report 10th and 90th percentiles.
